# Genomic and proteomic analyses of Nus-dependent non-lambdoid phages reveal a novel coliphage group prevalent in gut: mEp_immI_

**DOI:** 10.3389/fmicb.2025.1480411

**Published:** 2025-02-24

**Authors:** Honorio Negrete-Méndez, Guadalupe Valencia-Toxqui, Omar A. Sepúlveda-Robles, Emmanuel Ríos-Castro, Jairo C. Hurtado-Cortés, Victor Flores, Adrián Cázares, Luis Kameyama, Eva Martínez-Peñafiel, Fernando Fernández-Ramírez

**Affiliations:** ^1^Departamento de Genética y Biología Molecular, Centro de Investigación y de Estudios Avanzados del Instituto Politécnico Nacional (Cinvestav), México City, México; ^2^Department of Biology, Center for Phage Technology, Texas A&M University, College Station, TX, United States; ^3^Unidad de Investigación Médica en Genética Humana, UMAE Hospital de Pediatría, Centro Médico Nacional “Siglo XXI”, Instituto Mexicano del Seguro Social (IMSS), México City, México; ^4^Genomics, Proteomics and Metabolomic Core Facility (UGPM), LaNSE, Cinvestav-IPN, México City, México; ^5^Department of Biochemistry, University of Cambridge, Cambridge, United Kingdom; ^6^Parasites and Microbes Programme, Wellcome Sanger Institute, Cambridge, United Kingdom; ^7^Unidad de Genética, Hospital General de México “Dr. Eduardo Liceaga”, México City, México

**Keywords:** mEp_immI_ phages, phage phylogenomics, Nus-dependent phages, phage repressor, phage integration, antitermination, outer membrane receptors, phage superinfection-exclusion

## Abstract

**Introduction:**

Nus-dependent Mexican *Escherichia coli* phages (mEp) were previously isolated from clinical samples of human feces. Approximately 50% corresponded to non-lambdoid temperate phages integrating a single immunity group, namely immunity I (mEp_immI_), and these were as prevalent as the lambdoid phages identified in such collection.

**Methods:**

In this work, we present the structural and functional characterization of six representative mEp_immI_ phages (mEp010, mEp013, mEp021, mEp044, mEp515, and mEp554). In addition, we searched for homologous phages and prophages in the GenBank sequence database, and performed extensive phylogenetic analyses on the compiled genomes.

**Results:**

A biological feature-based characterization of these phages was carried out, focusing on proteins relevant to phage biological activities. This included mass spectrometry analysis of mEp021 virion structural proteins, and a series of infection assays to characterize the function of the main repressor protein and the lipoproteins associated with superinfection-exclusion; to identify the main host receptor proteins recognized by these phages and the prophage insertion sites within the host genome, which were associated with specific integrase sequence-types present in the viral genomes. Further, we compiled 42 complete homologous genomes corresponding to 38 prophages from E. coli strains and 4 phages from metagenomes, displaying a wide geographical distribution. Intergenomic distance analyses revealed that these phages differ from previously established phage clades, and whole-proteome similarity analyses yielded a cohesive and monophyletic branch, when compared to >5,600 phages with dsDNA genomes.

**Discussion:**

According to current taxonomic criteria, our results are consistent with a novel family demarcation, and the studied genomes correspond to 9 genera and 45 distinct species. Further, we identified 50 core genes displaying high synteny among the mEp_immI_ genomes, and these genes were found arranged in functional clusters. Furthermore, a biological feature-based characterization of these phages was carried out, with experiments focusing on proteins relevant to phage biological activities, revealing common traits as well as diversity within the group. With the integration of all these experimental and bioinformatics findings, our results indicate that the mEp_immI_ phages constitute a novel branch of *Caudoviricetes* distinct to other known siphovirus, contributing to the current knowledge on the diversity of phages infecting *Escherichia coli*.

## 1 Introduction

Bacteriophages or phages constitute the most abundant biological entity in our planet, with 10^31^ estimated viral particles in the biosphere, displaying a wide genetic diversity due to high recombination rates (Mushegian, 2020). Phage morphological features were one of the earliest criteria used for taxonomic classification, allowing the recognition of the order *Caudovirales* (i.e., tailed virus), which includes families *Myoviridae, Podoviridae* and *Siphoviridae* (Ackermann and Prangishvili, 2012; Mavrich and Hatfull, 2017). With the advent of second-generation sequencing methodologies, the number of phage and prophage genomes deposited in databases exponentially increased. Consequently, the morphology-based classification was revised in 2022, and genomic and proteomic features were incorporated as main taxonomic criteria, which in turn allow for a consistent and automated classification (Turner et al., 2021, 2024). Currently, 48 phage families have been integrated into a new class named *Caudoviricetes*, which includes all bacterial and archaeal tailed virus with icosahedral capsids and double-stranded DNA genomes (Turner et al., 2021, 2023; Zhu et al., 2022). Phages displaying substantial genome mosaicism may represent important challenges for taxonomic classification, as their genomes are the result of multiple and diverse horizontal transfer events that have occurred throughout their evolution, which have led to similarities in genome organization and regulation among phages from distinct origins (Hatfull, 2008; Cázares et al., 2014). Therefore, various unrelated phages were eventually clustered together in larger groups based on their biological features (Comeau et al., 2007; Evseev et al., 2021; Kupczok et al., 2022). Former terms such as Mu-like, T4-like, T7-like or lambdoid were traditionally used to designate groups of phages with functional resemblance, and refer to phages sharing similarities in genome organization, regulatory systems and protein functions, rather than a strict taxonomic identity based on nucleotide and protein sequence analyses (Comeau et al., 2007). For instance, phage P22 that infects *Salmonella* spp. is a podovirus (genus *Lederbergvirus*) morphologically different to phage λ (genus *Lambdavirus*), however, it is usually classified as a lambdoid phage, a large biological group mainly constituted by siphovirus infecting *Escherichia coli*. The similarities in genome organization among lambdoid phages have led to the ability to produce fertile hybrids (Botstein and Herskowitz, 1974; Campbell, 1994; Schicklmaier et al., 1999). Thus, the obtention of recombinant virions capable of producing viable progeny constitutes one basic genetic approach commonly used to discriminate between lambdoid and non-lambdoid coliphages; for this, co-infection assays using a reference lambdoid phage and a tested phage are performed. Other methods for phage grouping include the assessment of similarity in the N and Q antitermination systems, and in the repressor-based immunity control (Fattah et al., 2000; Hendrix, 2002; Degnan et al., 2007).

Lambdoid phages have been extensively characterized, being λ the prototype of this group. Their genomes are sequentially organized in functional clusters of conserved genes (regulation, replication, lysis, structural proteins, etc.), with several interspersed accessory genes (Casjens and Hendrix, 2015). The gene regulation of these phages is organized in four main transcriptional units: the left operon which includes the antitermination *N* gene, among others; the right operon with early regulators *cro* and *c*II, and replication genes such as *O* and *P*; the late operon involved in transcription of structural and lysis genes; and the immunity operon containing the *c*I repressor gene, and others (Dydecka et al., 2020; Kupczok et al., 2022).

One particular trait of lambdoid phages is the requirement of host Nus factors and the viral N and Q proteins for their antitermination function. Kameyama et al. (1999) reported a collection of 96 Nus-dependent coliphages named mexican *Escherichia coli* phages (mEp), which were originally isolated from clinical samples of human feces. From these, 46 corresponded to lambdoid phages that were further classified in 19 immunity groups (II-XX). The remaining 50 phages integrated the most numerous immunity group (I) which displayed no similarity to lambdoid phages, other than the use of host Nus factors. Conversely, the DNA of these phages did not hybridize with λ DNA probes; their structural proteins (i.e., those related to the capsid and tail viral structures) were not recognized by anti-λ antibodies, most were not induced by UV light treatment, and no viable progeny was obtained in co-infection assays using λ-BLK20, a phage λ derivative harboring a *N-Iac*Z gene fusion at the left side of *att* site that produces faint-blue lysis plaques on standard X-gal plates, allowing the screening of recombination events between phages (Kameyama et al., 1991). All these results indicated that the mEp immunity group I phages (mEp_immI_) were distinct from the lambdoid phages. Noteworthy, these phages were as prevalent in biological samples as the lambdoid groups (Kameyama et al., 1999, 2001).

In this work, we extended the characterization of the mEp_immI_ group by experimental approaches and whole-genome sequencing of 6 representative coliphages (12%), including mEp010, mEp013, mEp021, mEp044, mEp515 and mEp554. We also identified 42 homologous complete phage and prophage genomes from sequences deposited in the GenBank database. Following current taxonomic criteria, we present extensive comparative genomics and proteomics analyses, revealing that the mEp_immI_ group constitutes a novel branch of *Caudoviricetes* distinct from previously described coliphages. In order to explore their similarities and diversity, and reinforce their status as an independent group, we performed a traditional biological-feature characterization of these phages, focusing on proteins whose function is related to the virion structure as well as to the most relevant biological activities of phages, namely receptor usage and recognition, genome integration, lysogeny regulation, antitermination, and superinfection-exclusion. Taken together, our results provide evidence of the uniqueness and diversity within this novel phage group.

## 2 Materials and methods

### 2.1 Bacteria, plasmids, and media

The bacterial strains and plasmids used in this study are listed in Supplementary Table S1. Derivative *E. coli* K-12 wild type strain W3110 was used for phage propagation and infection assays. Keio strains JW0940-6 (*omp*A^−^), JW3996-1 (*lam*B^−^) and JW2203-1 (*omp*C^−^) were used in infection assays in order to assess the different membrane receptor proteins used by the studied phages (Datsenko and Wanner, 2000; Baba et al., 2006). The DH5α strain was used for plasmid manipulation. Lysogenic broth (LB; 10 g/L tryptone, 5 g/L yeast extract 10 g/L NaCl) and TMG phage-dilution media (10 mM Tris–HCl, pH 7.2, 10 mM MgSO_4_, and 0.1% gelatin) were prepared as previously described (Silhavy et al., 1984). LB media was supplemented with ampicillin (100 μg/mL), kanamycin (30 μg/mL) or isopropyl β-D-1-thiogalactopyranoside (IPTG 0.1 mM) (Sigma-Aldrich, St. Louis, MA, USA) when required. All media were purchased from BD Difco™ (Franklin Lakes, NJ, USA). T4 DNA ligase and restriction endonucleases *Nde*I, *EcoR*I, and *Hin*dIII were purchased from New England Biolabs (Ipswich, MA, USA).

### 2.2 Phages and prophages

Phages mEp021, mEp010, mEp013, mEp044, mEp515 and mEp554 were selected as representative phages from the immunity group I (Kameyama et al., 1999). For the experimental approaches described in this study, the phages are listed in Supplementary Table S1. The λ phage was used as negative control in all the infection assays. The phages and prophages used for comparative genome analyses are listed in Table 1. These genomes were retrieved from the GenBank database (NCBI) through BLASTn searches (Altschul et al., 1997) using as query a phage genome belonging to immunity group I (mEp021). We selected hits with query coverage threshold = 60%, *E*-value = 0.0, and percent identity threshold = 70%. For phage genomes (*n* = 4), the keywords “Viruses (taxid:10239)” or “Caudoviricetes (taxid:2731619)” were used in the query, and for prophage genomes (*n* = 38) the keyword was “*Escherichia coli* (taxid:562).” In all cases, the retrieved genomes were manually inspected to assure their completeness. Genomes corresponding to 11 siphovirus (λ, HK022, HK97, N15, T1, T5, psiM2, SPBc2, c2, phi-C31, and L5) and 1 podovirus (P22) were used as external references in the main comparative analyses (Supplementary Table S2), and an additional larger dataset, including these reference phages and 50 additional neighboring phages (as observed in the ViPTree analysis, Supplementary Figure S4A), was used to increase the coverage of our analyses (Supplementary Table S3).

**Table 1:** Studied phages and prophages of mEp_immI_ group.

mEp (our laboratory)	Host	Bacterial host	Isolation source	Country	Accession number
mEp021	*Homo sapiens*	*Escherichia coli*	Feces	México	MH706966.1
mEp010	*Homo sapiens*	*Escherichia coli*	Feces	México	PP180001
mEp013	*Homo sapiens*	*Escherichia coli*	Feces	México	PP180002
mEp044	*Homo sapiens*	*Escherichia coli*	Feces	México	PP180004
mEp515	*Homo sapiens*	*Escherichia coli*	Feces	México	PP180003
mEp554	*Homo sapiens*	*Escherichia coli*	Feces	México	PP180005

Bacteriophages (metagenomes)	Host	Bacterial host	Isolation source	Country	Accession number
0621_18038	*Homo sapiens*	ND	Feces	Japan	OP073365.1
ct11k1	*Homo sapiens*	ND	Human gut	Denmark	BK025398.1
ctPXR1	*Homo sapiens*	ND	Human gut	USA	BK024291.1
ctx8n3	*Homo sapiens*	ND	Human gut	Finland	BK020724.1

Prophages (bacterial genomes)	Host	Bacterial host	Isolation source	Country	Accession number
FHI99	*Homo sapiens*	*Escherichia coli*	Feces	Norway	LM997270.1
UMNK88	*Sus scrofa domesticus*	*Escherichia coli*	Porcine Neonatal Diarrhea	USA	NC_017641.1
504005_aEPEC	*Homo sapiens*	*Escherichia coli*	Feces	India	NZ_CYBZ01000006.1
09–00049	ND	*Escherichia coli*	Lettuce	USA	NZ_CP015228.1
Ecol_244	*Homo sapiens*	*Escherichia coli*	ND	Argentina	CP019020.1
1–110-08_S4_C1	*Homo sapiens*	*Escherichia coli*	Feces	Tanzania	NZ_JHDK01000106.1
1–392-07_S4_C3	*Homo sapiens*	*Escherichia coli*	Feces	Tanzania	NZ_JOSH01000199.1
HVH 69 (4–2837072)	*Homo sapiens*	*Escherichia coli*	Blood (UTI induced bacteremia)	Denmark	AVUV01000007.1
HVH 103 (4–5904188)	*Homo sapiens*	*Escherichia coli*	Blood (UTI induced bacteremia)	Denmark	AVVS01000012.1
KOEGE 62 (175a)	*Homo sapiens*	*Escherichia coli*	Blood (UTI induced bacteremia)	Denmark	AWAL01000002.1
KTE83	*Homo sapiens*	*Escherichia coli*	ND	Denmark	ANUT01000018.1
MS21-1	*Homo sapiens*	*Escherichia coli*	Gastrointestinal_tract	USA	NZ_GG772625.1
FBPI	*Sus scrofa domesticus*	*Escherichia coli*	Feces	The Netherlands	AYKC01000020.1
O5:K4(L):H4 str. ATCC235002	*Homo sapiens*	*Escherichia coli*	Urine	NA	NZ_CAPL01000001.1
G180	*Bos taurus*	*Escherichia coli*	Mastitis	France	NZ_LONT01000016.1
HVH 110 (4–6978754)	*Homo sapiens*	*Escherichia coli*	Blood (UTI induced bacteremia)	Denmark	AVVY01000006.1
UMEA 3323–1	*Homo sapiens*	*Escherichia coli*	Urine	Sweden	NZ_KI530679.1
MIN12	*Homo sapiens*	*Escherichia coli*	Human postoperative wound swab	Poland	CP069657.1
LSU61	*Cervidae*	*Escherichia coli*	ND	USA	CP038336.1
2–331	ND	*Escherichia coli*	Sewage	Norway	CP110117.1
127	*Canis lupus familiaris*	*Escherichia coli*	Whole organism	United Kingdom	CP023377.1
190	*Homo sapiens*	*Escherichia coli*	Urine	USA	CP020520.1
C16EC0292	*Homo sapiens*	*Escherichia coli*	Blood (bloodstream infection)	South Korea	CP088668.1
E21845	*Homo sapiens*	*Escherichia coli*	Diarrhea	Thailand	NZ_CP076272.1
FAH	*Homo sapiens*	*Escherichia coli*	Feces from healthy subject	China	CP116035.1
GN02175	*Homo sapiens*	*Escherichia coli*	Blood (bloodstream infection)	USA	CP041550.1
GN04592	*Homo sapiens*	*Escherichia coli*	Blood (bloodstream infection)	USA	CP095538.1
GN05505	*Homo sapiens*	*Escherichia coli*	Blood (bloodstream infection)	USA	CP095526.1
KKa019	ND	*Escherichia coli*	River water	Japan	CP091664.1
M1/5	*Homo sapiens*	*Escherichia coli*	Feces	Germany	CP053296.1
MLI107	*Homo sapiens*	*Escherichia coli*	Feces	Mali	CP116987.1
PAR	*Cacatuidae*	*Escherichia coli*	Feces	China	CP012379.1
RHBSTW-00392	ND	*Escherichia coli*	Wastewater effluent sample	United Kingdom	CP056564.1
RM9245	ND	*Escherichia coli*	Feces	USA	CP044314.1
STEC306	*Ovis aries*	*Escherichia coli*	Mutton	China	NZ_CP091016.1
STEC307	*Ovis aries*	*Escherichia coli*	Mutton	China	NZ_CP091018.1
STEC308	*Homo sapiens*	*Escherichia coli*	Patient	China	NZ_CP041435.1
WCHEC025970	*Homo sapiens*	*Escherichia coli*	Pure culture	China	CP036177.1

Six mEp_immI_ phages are from the collection previously reported (Kameyama et al., 1999), and were experimentally characterized in this work. Four homologous bacteriophage and 38 prophage complete genomes were identified from metagenomes and *E. coli* genomes from GenBank, respectively, and were integrated in subsequent phylogenomics analyses.

### 2.3 Phage propagation, phage DNA extraction and RFLP analysis

For phage propagation, 1 mL of *Escherichia coli* W3110 (Bachmann, 1972; Asakura and Kobayashi, 2009) was cultured overnight (O/N) and mixed with ∼10 plaque-forming units (PFU) of phage, and 1 mL of 0.5 M CaCl_2_:MgCl_2_ (1:1). After allowing the phages to adsorb for 15 min, 50 mL of LB broth was added to obtain a final concentration of 10 mM CaCl_2_:MgCl_2_ (1:1). The culture was incubated at 37°C with shaking at 200 rpm until the cells were lysed, 5 mL of chloroform was added, the sample was centrifuged at 4,200 × *g* for 10 min, and the supernatant was recovered. For phage DNA extraction, 50 mL of supernatant was treated with PEG-8000 (Sigma-Aldrich, St. Louis, MA, USA) and NaCl at final concentration of 10% and 1 M, respectively. The solution was incubated for 8 h at 4°C and centrifugated at 10,590 × *g* for 10 min. The obtained pellet was resuspended in 1 mL of TMG, treated with chloroform (v:v) (J.T. Baker, Phillipsburg, NJ, USA) and centrifuged at 9,300 × *g* in order to eliminate PEG-8000 (Sigma-Aldrich, St. Louis, MA, USA). The recovered phages were purified by cesium chloride gradient as indicated in previous protocols (Shevchenko et al., 2006). The collected band was dialyzed in Tris 50 mM, NaCl 10 mM and MgCl_2_ 10 mM solution for 12–18 h at 4°C. The phage DNA was extracted as described previously (Sambrook et al., 1989) and the pellet was resuspended in 50 μL of nuclease-free water for restriction analysis and DNA sequencing. For restriction fragment length polymorphism (RFLP) analysis, 1 μg of genomic DNA was restricted with *Nde*I (10 U), then the products were resolved and visualized in 1% agarose gel electrophoresis.

### 2.4 Transmission electron microscopy (TEM)

10 μL of dialyzed CsCl-purified bacteriophages (1×10^11^ PFU/mL) were deposited on a Formvar carbon-coated grid and incubated for 1 min. The excess liquid was removed with filter paper and then 10 μL of 1% uranyl acetate (Electron Microscopy Science, Hatfield, PA, USA) was applied for 30 and 60 s and removed. The preparations were analyzed using the electron transmission microscopes JEM-1400 (JEOL, Akishima, Japan) at 100 kV.

### 2.5 Virion structural protein analysis and detection by mass spectrometry (MS)

#### 2.5.1 Sample processing of the structural proteins of the virus

30 μL of CsCl-purified bacteriophage particles were resuspended in Laemmli buffer and were boiled in a water bath for 10 min followed by 5 min on ice. The sample (20 μL) was resolved by 16% SDS-PAGE at 90 V for 2.5 h. Protein bands were visualized by staining with Silver Stain Plus (Bio-Rad, Hercules, CA, USA), following the protocol provided by the supplier. Proteins from a mEp021 virion sample were resolved by 10% SDS-PAGE. Fifteen gel slides corresponding to discrete protein bands were enzymatically digested with trypsin, according to the modified protocol of Shevchenko et al. (2006) and Barrera-Rojas et al. (2023). Briefly, bands were excised from the gel and transferred into centrifuge microtubes to be destained using a solution containing 2.5% formic acid (FA), 50% methanol (MeOH), and were subsequently dehydrated with acetonitrile (ACN), and the remaining solvent was eliminated in a Savant DNA120 SpeedVac Concentrator (Thermo Fisher Scientific, Waltham, MA, USA) for 10 min. Then, proteins were reduced with 10 mM DTT (Sigma-Aldrich, St. Louis, MO, USA) in 100 mM ammonium bicarbonate (ABC) (Sigma-Aldrich, St. Louis, MO, USA) and alkylated with 50 mM iodoacetamide (IAA) (Sigma-Aldrich, St. Louis, MO, USA) in 100 mM ABC. Afterward, bands were washed with 100 mM ABC and dehydrated with ACN; subsequently, bands were hydrated and washed again with 100 mM ABC and dehydrated with ACN. Then, the excess solvent was removed using the SpeedVac for 10 min. Proteins were enzymatically digested overnight using 20 ng/μL of trypsin (Sigma-Aldrich, St. Louis, MA, USA) in 50 mM ABC at 37°C in a Precision water bath (Thermo Fisher Scientific, Waltham, MA, USA). Once the time had passed, the reaction was stopped with 40 μL of a solution of 5% FA for 10 min at room temperature; subsequently, peptides were eluted from the gel for two cycles using 40 μL of a solution of 5% FA and 50% ACN. Peptides were concentrated in the SpeedVac and desalted using ZipTips C18 (Millipore, Burlington, MA, USA).

#### 2.5.2 Mass spectrometry analysis

The resulting tryptic peptides were concentrated to an approximate volume of 10 μL; 4 μL were loaded into a Symmetry C18 Trap V/M precolumn (Waters, Milford, MA, USA); 180 μm X 20 mm, 100 Å pore size, 5 μm particle size and desalted using as a mobile phase A, 0.1% formic acid (FA) in H_2_O and mobile phase B, 0.1% FA in acetonitrile (ACN) under the following isocratic gradient: 95% mobile phase A and 5% of mobile phase B at a flow of 15 μL/min during 5 min. Then, peptides were loaded and separated on a HSS T3 C18 Column (Waters, Milford, MA, USA); 75 μm X 150 mm, 100 Å pore size, 1.8 μm particle size; using an UPLC ACQUITY M-Class (Waters, Milford, MA, USA) with the same mobile phases mentioned above under the following gradient: 0–3 min 10% B (90% A), 15 min 20% B (80% A), 60 min 60% B (40% A), 61–64 min 90% B (10% A), 65 to 70 min 10% B (90% A) at a flow of 250 nL/min and 30°C. The spectra data were acquired in a mass spectrometer with electrospray ionization (ESI) and ion mobility separation (IMS) Synapt G2- S*i* (Waters, Milford, MA, USA) using data-independent acquisition (DIA) through HDMS^E^ mode (Full-Scan DIA) (Lou and Shui, 2024). The tune page for the ionization source was set with the following parameters: 2.60 kV in the sampling capillary, 30 V in the sampling cone, 30 V in the source offset, 70°C for the source temperature, 0.6 Bar for the nano flow gas and 120 L/h for the purge gas flow. Two chromatograms were acquired (low and high energy) in positive mode in a *m/z* range of 50–2000 with a scan time of 500 ms. No collision energy was applied to obtain the low energy chromatogram, while for the high energy chromatograms the precursor ions were fragmented in the transfer using a collision energy ramp of 30–45 V. Synapt G2-S*i* was calibrated with [Glu^1^]-Fibrinopeptide fragments, through the precursor ion [M + 2H]^2+^ = 785.84261 fragmentation of 32 eV with a result of ≤1 ppm across all MS/MS measurements.

#### 2.5.3 Database search

The generated mEp021.raw files containing MS and MS/MS spectra were deconvoluted and compared using ProteinLynx Global SERVER (PLGS) (Li et al., 2009) v3.0.3 software (Waters, Milford, MA, USA) using a target decoy strategy against an in-house fasta database of 82 predicted proteins from the mEp021 genome (Elias and Gygi, 2009; Käll et al., 2007). Workflow parameters were Trypsin as a cut enzyme and one missed cleavage allowed: carbamidomethyl (C) as a fixed modification and acetyl (K), acetyl (N-term), amidation (N-term), deamidation (N, Q), oxidation (M), Phosphoryl (S, T, Y) as variable modifications. Automatic peptide and fragment tolerance, minimum fragment ion matches per peptide: 2, minimum fragment ion matches per protein: 5, minimum peptide matches per protein: 1, and false discovery rate of 4%.

### 2.6 Genome sequencing and *de novo* assembly of mEp021

The genome of prototype phage mEp021 was sequenced at the National Laboratory of Genomics for Biodiversity (Cinvestav, Irapuato, México) using the AB SOLiD technology (Applied Biosystems, Waltham, MA, USA). The sequencing reads were preprocessed for base calling, quality score assignment and filtering using the Applied Biosystems (Waltham, MA, USA) de novo assembly accessory software with default values. The phage genome was assembled de novo using Velvet v1.1 (Zerbino and Birney, 2008) with default settings, resulting in eight contigs. The assembly was examined using Tablet (Milne et al., 2015) and manually edited to remove errors. The complete mEp021 genome was then assembled into a single scaffold by Sanger sequencing, using primers designed to amplify outwards of each contig in order to fill the sequence gaps (see Supplementary Table S4 and Supplementary Figure S1). Sequencing products were read in an ABI PRISM 310 automatic sequencer (Applied Biosystems, Waltham, MA, USA) of the Genetics and Molecular Biology Department facility (Cinvestav-IPN, México City, México).

Coding sequences in the genome of mEp021 were predicted with heuristic Hidden Markov Models using GeneMark v4.3 (Borodovsky and Lomsadze, 2011), and the corresponding ribosome-binding sites were detected with RBS_finder (Suzek et al., 2001). Gene calling was carried out via comparison of the predicted mEp021 ORF products against the non-redundant protein database from NCBI using BLASTp, with a 50% coverage, 1E-4 e-value and > 0% identity threshold (Altschul et al., 1997). InterProScan (Paysan-Lafosse et al., 2022) was used to identify conserved domains and protein families among the identified ORFs. The Artemis annotation tool (Carver et al., 2011) was used to integrate the results from BLASTp and InterProScan, and to visualize the genome. Final annotation was achieved by comparing these annotations with the one obtained through the PHAge Search Tool (PHAST) server (Zhou et al., 2011).

### 2.7 Genome sequencing, assembly, and annotation of mEp010, mEp013, mEp044, mEp515, mEp554

Phage genomes were sequenced using Illumina technology, with services provided by the Microbial Genome Sequencing Center (Pittsburgh, PA, USA) using a MiSeq instrument (for: mEp010, mEp013, mEp044, mEp554) and in the SeqCenter (Pittsburgh, PA, USA) using a NovaSeq 6000 sequencer (for: mEp515). In all cases, the sequencing was based on the protocol by Baym et al. (2015). The genome assembly was performed using the Galaxy interface,^1^ a graphic environment that provides well established bioinformatics tools for genome assembly and analyses. The quality of the raw reads was evaluated with FastQC version 0.12.1.^2^ Low quality bases were removed using Trimmomatic version 0.38.1 using the default parameters (Q20), and the reads were assembled into contigs using the Shovill genome assembly pipeline, which is dedicated to microorganisms with small genome sizes. This pipeline uses the SPAdes assembler version 1.1.0 at its core, which is currently considered as the standard *de novo* genome assembler for Illumina platforms; K-mer was set as default. Once assembled, the complete genomes were annotated using the Genome Annotation Service available in the Bacterial and Viral Bioinformatics Resource Center (BV-BRC),^3^ using the following parameters: annotation recipe: “bacteriophages”; taxonomy name: “Enterobacteria phage mEp021” (the mEp021 genome was set as reference), and Taxonomy ID: “1150757.” The annotation recipe for bacteriophages is based on the PHANOTATE method, which is specifically designed for phage gene calling (McNair et al., 2019). The accession numbers of mEp010, mEp013, mEp021, mEp044, mEp515 and mEp554 are, respectively, indicated in Table 1.

### 2.8 *In silico* comparative phylogenomics

Two complementary approaches were used to analyze the phylogenetic relationships of the studied mEp_immI_ phages: one at the nucleotide sequence-level, including whole-genome alignments and distance-based clustering using different tools, and another at the whole proteome-level; in both cases we included sets of bacteriophage genomes as external references. Whole-genome alignments were performed using the NGPhylogeny server (Lemoine et al., 2019), which includes the Multiple Alignment Program for amino acid or nucleotide sequences (MAFFT), the Block Mapping and Gathering with Entropy program (BMGE) for aligned sequence cleaning, and Maximum likelihood-based Inference of phylogenetic trees with Smart Model Selection (PhyML+SMS), to construct intergenomic distance-based phylogenetic trees. The VIRIDIC tool (Moraru et al., 2020) was used to calculate intergenomic distance matrices to allow the identification of genera and species within the analyzed phages.

The Viral Proteomic Tree server (ViPtree) v4.0 (Nishimura et al., 2017), which is a tBLASTx-based tool recommended by International Committee on Taxonomy of Viruses (ICTV) for family-level classification of viruses (Turner et al., 2024), was used to perform comparative whole-proteome analysis of the 48 mEp_immI_ phages against the proteomes of >5,600 dsDNA reference phages deposited in its database.^4^ VirClust, a tool for higher taxon-level analyses (Moraru, 2023), was used to calculate intergenomic distances based on conserved protein clusters (PC) and to identify viral genome clusters (VGC), using 100 bootstrap resampling and default parameters for the 60 genomes dataset, and a 0.7 distance threshold for the 110 genomes dataset.

Analyses of the repressor, integrase, Gp17-like, J and Lpp putative proteins of the mEp_immI_ phages were carried out using MultAlin^5^ for multiple protein sequence alignments. Analysis of the sequences surrounding the *att*R and *att*L of the prophages were carried out through multiple nucleotide-sequence alignments, which were performed with Clustal Omega (v1.0)^6^ (Sievers et al., 2011). All of these alignments were visualized using ESPript (v3.0) (Robert and Gouet, 2014). For the analysis of *nut* sites, the sequences of nutR1, nutR2 and nutL from mEp021 were searched in the genomes of the other mEp_immI_ phages, and the consensus sequence was visualized using WebLogo v2.8.2 (Crooks et al., 2004).

### 2.9 Identification of the core genome of the mEp_immI_ phages

We manually determined the percentages of core genes, flexible genes and unique genes in the set of 48 phages and coliphages genomes. In order to homogenize gene annotations, the 42 complete genome sequences of the homologous phages and prophages from GenBank and mEp021 were reannotated, using PHANOTATE via BV-BRC (for details see the previous subsection). According to the relative position of the genes identified in the phage genomes, all of the predicted proteins were manually inspected. Those sharing similar amino acid sequences in either terminal segment, were selected and pairwise-aligned against the corresponding mEp021 proteins; this allowed the verification of their identities, which in all cases was above 58%. In this manner, we identified gene products that were common to all the studied genomes (i.e., core genes); gene products that were identified in more than one genome but not all genomes were considered as flexible, and those only present in a single genome were labeled as unique. We performed pairwise-alignments of the core proteins, taking their corresponding mEp021 homologs as reference, using Geneious Prime® 2024.0.5. The resulting identity percentage scores were manually compiled and used to generate a correlation heatmap using ChiPlot.^7^ Automated analysis of the core genome of the 48 mEp_immI_ phages was performed using VirClust with default parameters (Moraru, 2023); the gene calling pipeline in this tool is based on the MetaGeneAnnotator for bacterial and phage genes (Noguchi et al., 2008), and allows the identification of conserved protein clusters among the input phage genomes.

### 2.10 Construction of plasmids pRep_021_, pLpp_021_ and pLpp_010_

The biological function of the predicted repressor (Rep) and lipoprotein (Lpp) proteins of the mEp_immI_ phages was tested by means of phage infection assays. For this, we used our sequenced phages as templates to amplify and clone the corresponding coding genes in expression vectors. Considering the strong sequence similarity that the repressor proteins displayed among all the mEp_immI_ phages, we selected the repressor gene of the archetype phage mEp021, which is the best characterized phage of our collection. Hence, the *gp15* repressor gene of mEp021 was amplified using specific primers (Supplementary Table S4). For lipoprotein genes, we based our selection on the following criteria: first, two out of the four identified lipoprotein sequence-types were represented in the phages of our collection (mEp021 and mEp515 for type-A lipoproteins, and mEp010, mEp013, mEp044 and mEp554 for type-C lipoproteins); notably, the amino acid sequences of the type-A lipoproteins were identical, while the ancestral sequence for type-C lipoproteins corresponded to that of mEp010. Hence, the *gp81* gene of mEp021 and the *gp116* gene of mEp010 were amplified via PCR using the primers described in Supplementary Table S4. In the case of *gp81*, the reverse primer was designed with a TWA codon (W = A or T) positioned upstream to the sequence coding for the 6xHisTag, and a TAA codon after it (Supplementary Figure S2). For the experiments described in this work, we only selected the construction with a stop codon before the 6xHisTag, which yielded a wild-type Gp81 lipoprotein. Amplicons were inserted into the transit plasmid pJET1.2blunt (Thermo Fisher Scientific, Waltham, MA, USA). Subsequently, the inserts corresponding to *gp15*, *gp81* and *gp116* genes were recovered from the transit plasmid by digestion with *Eco*RI and *Hin*dIII and were ligated into the *Eco*RI–*Hin*dIII sites of the low copy-number vector pKQV4 (Strauch et al., 1989) for IPTG-inducible expression from the P*tac* promoter, generating the plasmids pRep_021_, pLpp_021_, and pLpp_010_ (Supplementary Figure S2).

### 2.11 Phage infection assays

Several aspects related to the mEp_immI_ most relevant biological activities were tested by means of phage infection assays, including the repressor function, outer membrane receptor (OMR) recognition, and superinfection-exclusion. Infection assays were performed as described previously (Arguijo-Hernández et al., 2018). For each case, Keio collection strains and W3110 were transformed with pRep_021_, pLpp_021_, pLpp_010_ or pKQV4, respectively. Cultures of each strain were grown O/N in LB broth at 37°C in a rotatory shaker at ∼200 rpm, and 300 μL of each one were mixed with 3 mL of soft top agar and poured on LB-agar plates, forming a bacterial lawn. Serial dilutions of phages were prepared in TMG solution and spotted onto the bacterial lawn, and the plates were incubated overnight at 37°C.

### 2.12 *In silico* three-dimensional structure analyses of the repressor proteins

Three-dimensional models of the repressor protein of mEp021 (namely Rep_021_) were generated using the ColabFold software (Mirdita et al., 2023) available in UCSF ChimeraX v1.7.1 (Meng et al., 2023), the DI-TASSER (Deep learning-based Iterative Threading ASSEmbly Refinement) server (Zheng et al., 2023) and the Phyre^2^ Protein Homology/analogY Recognition Engine V 2.0 server (Kelley et al., 2015). The crystallographic structure of the λ repressor (3BDN) was used for further superposition analysis with the different Rep_021_ models, using Phyre^2^ and PyMOL (Schrödinger and DeLano, 2020).

### 2.13 Phage attachment site validation by PCR and sequencing

During the lysogenic cycle of phages, the phage DNA may integrate into the host genome, using an integrase protein that recognizes specific insertion motifs within the host genome and promotes the integration of the phage DNA. In order to explore the integration process of the mEp_immI_ phages, we predicted the attachment sites and integrase types through sequence analyses, and validated these findings in the W3110 (mEp021) lysogen by means of DNA sequencing. Briefly, bacterial colonies from W3110 (mEp021) lysogens and the W3110 isogenic strain were selected and resuspended in 100 μL of distilled H_2_O. Genomic DNA of these cells was obtained by mechanical disruption using glass beads (SIGMA, G-1634) and vortexing for 5 min. Then, 5 μL of each lysate were mixed with 10 nM dNTP mix, and 10 μM primers T1, B1, T2 and B2 in different paired combinations (Supplementary Table S4), and Dream Taq polymerase (Thermo Fisher Scientific, Waltham, MA, USA), according to the PCR protocol provided for the enzyme. Reactions were performed using a T100 Thermal Cycler (Bio-Rad, Hercules, CA, USA) under the following conditions: a denaturing step at 94°C/5 min, followed by 35 amplification cycles of 94°C/30 s, 58°C/60 s and 68°C/30 s for each cycle, and a final extension step at 72°C/10 min. The PCR products were then sequenced using the *att*R and *att*L primers (described in Supplementary Table S4), in an automatic sequencer Perkin Elmer™ ABI PRISM™ 310 (Applied Biosystems, Waltham, MA, USA), at the sequencing facility of the Genetics and Molecular Biology Department (Cinvestav-IPN, México City, México).

### 2.14 Verification of Keio mutant strains

The genotypes of the JW0940-6, JW3996-1 and JW2203-1 Keio strains were verified by PCR (Supplementary Figure S9), using specific primers listed in Supplementary Table S4. Briefly, the forward primer was directed to the middle region of the kanamycin cassette and the reverse primer was directed to the 3′ region of the *omp*A, *lam*B or *omp*C genes, respectively. The bacterial DNA was obtained by mechanical disruption with glass beads (Sigma-Aldrich, St. Louis, MA, USA) and vortexing for 5 min. The PCR reaction mix was prepared following the protocol supplied for DreamTaq DNA polymerase (Thermo Fisher Scientific, Waltham, MA, USA). Reactions were performed using a T100 Thermal Cycler (Bio-Rad, Hercules, CA, USA) under the following conditions: a denaturation step for 5 min at 95°C, followed by 34 cycles of denaturation for 45 seg at 95°C, annealing for 30 seg at 56.5°C and elongation for 60 seg at 68°C, and a final step of 7 min at 68°C. The amplification products were resolved by electrophoresis in 1% agarose gels stained with ethidium bromide, and visualized using a UV transilluminator.

## 3 Results

### 3.1 Structural characterization of representative mEp_immI_ phages

**Figure 1:**
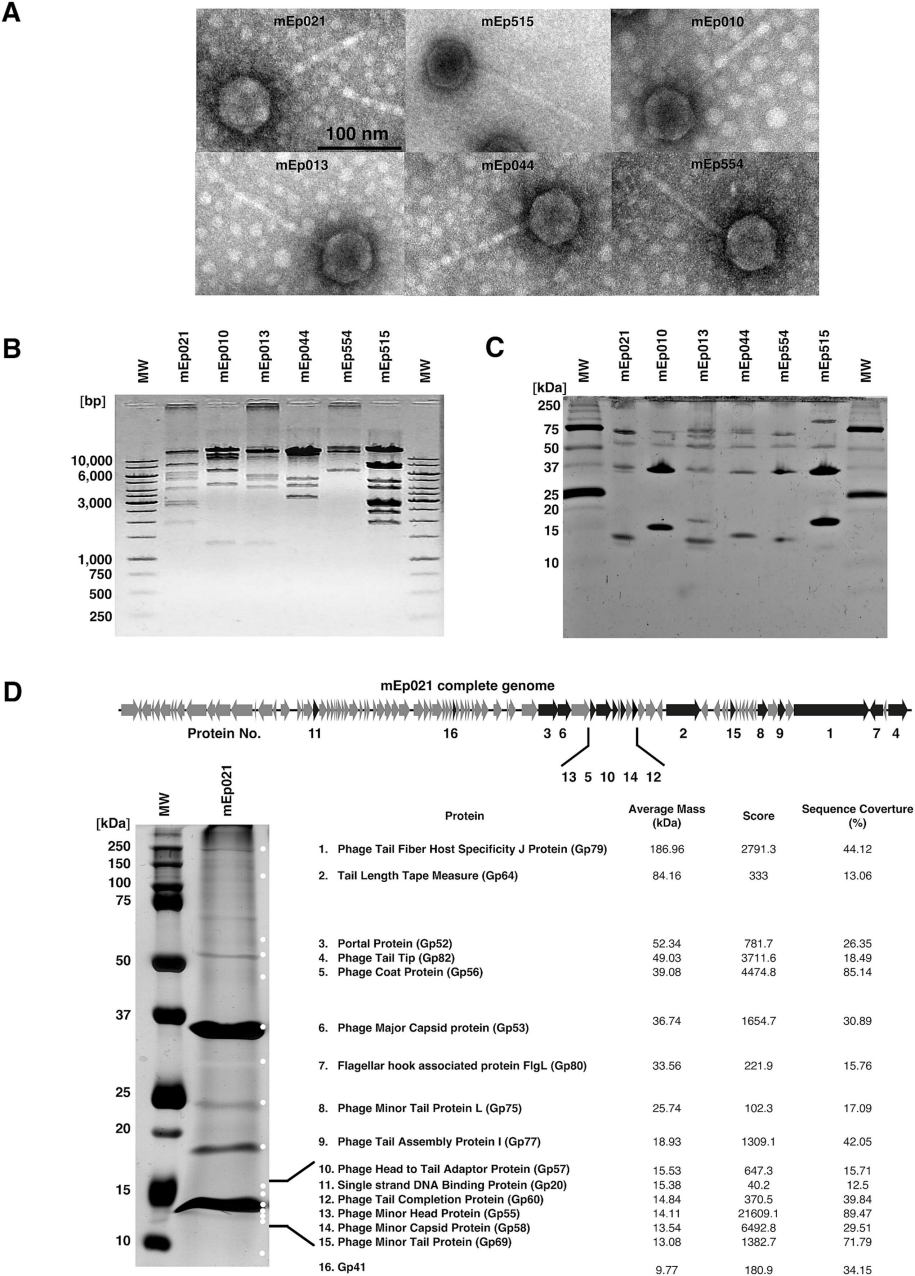
Structural characterization of six representative phages of the mEp_immI_ group. **(A)** Transmission electron microscopy (150x magnification) revealed ~60 nm phage particle sizes. **(B)** RFLP analysis using *Nde*I indicated genomic differences among the studied phages. **(C)** Structural proteins of variable size were observed among the 6 phages, in total virion proteins 16% SDS-PAGE analysis. **(D)** Sixteen discrete protein bands were identified from the 10% SDS-PAGE analysis of the mEp021 structural proteins by HPLC/MS/MS. These proteins were subsequently mapped in the viral genome, which is illustrated at the top of the figure.

**Figure 2:**
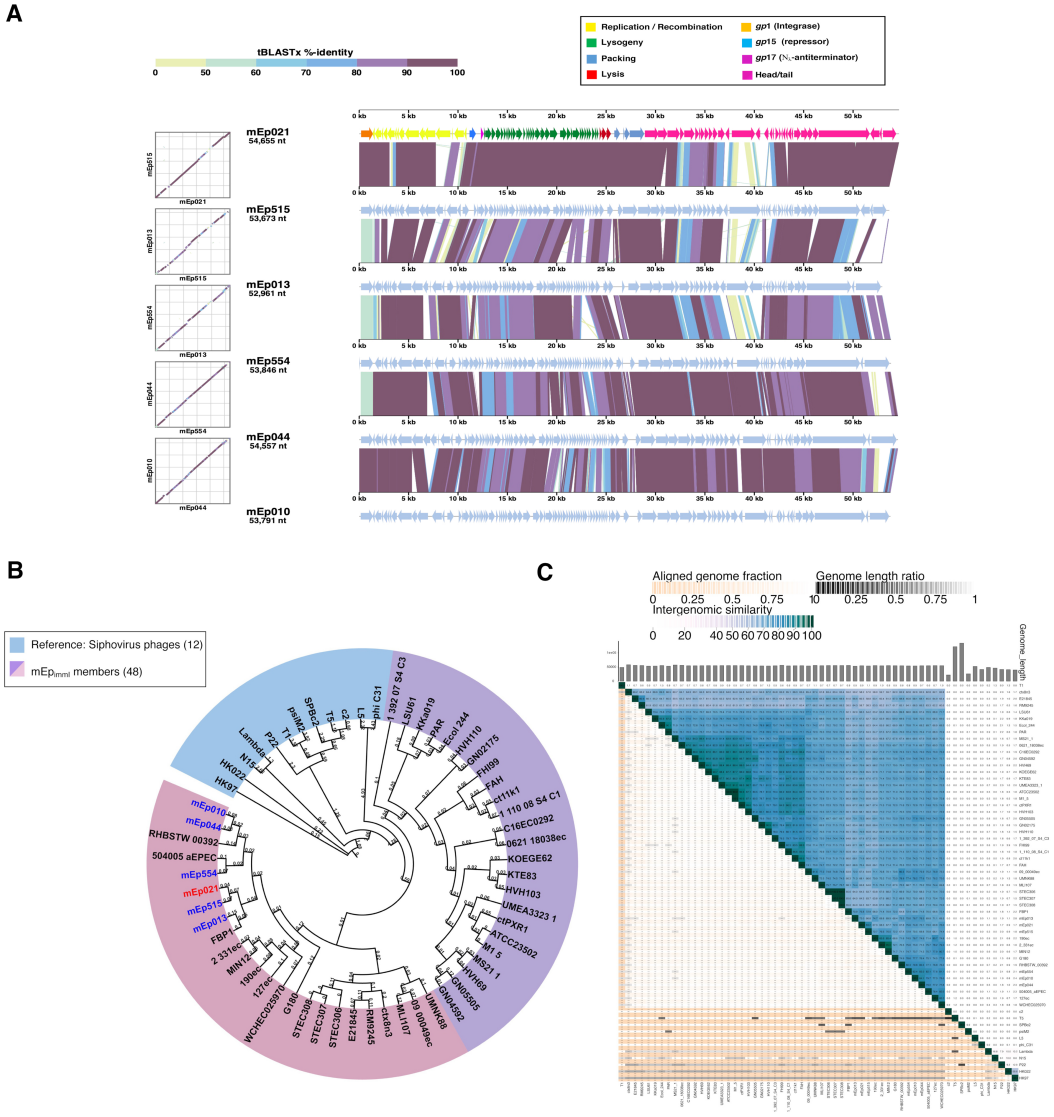
Comparative phylogenomic analysis of the mEp_immI_ group. **(A)** tBLASTx genome alignment of the 6 mEp_immI_ representative phages, showing conserved functional clusters of genes; the proteins that were experimentally characterized in this work are represented by orange, blue and pink arrows. **(B)** Whole-genome sequence analysis indicates that the mEp_immI_ phages cluster apart from reference siphovirus. Genomes were aligned to construct a distance-based phylogenetic tree using the NGPhylogeny server; the 48 studied phages described in Table 1 were included, as well as twelvedifferent dsDNA siphovirus as external reference group (blue background); two main subgroups of mEp_immI_ phages were observed in this analysis (indicated in purple and pink background, respectively). **(C)** VIRIDIC heatmap representing the intergenomic similarity values in blue color gradient (right section, where darker tones indicate higher similarity values). The 48 mEp_immI_ genomes and 12 external reference phage genomes were included in the analysis. The orange color gradient represents the aligned genome fractions (left section), where darker colors correspond to low values (i.e., only a small fraction of the genomes were aligned). Bars at the top of the figure represent the respective genome lengths, and ratios of each aligned pairs are indicated in a gray gradient, within the left area of the heatmap.

We experimentally characterized six mEp_immI_ phages that were selected from the collection previously reported by Kameyama et al. (1999): mEp010, mEp013, mEp021, mEp044, mEp515, and mEp554. These phages present a siphovirus morphology, with ~60 nm capsids and ~130 nm tails, according to TEM (Figure 1A). Unique RFLP and structural protein electrophoretic patterns were observed for each of these phages (Figures 1B,C). In the case of mEp021, we identified 16 structural proteins by mass spectrometry (MS) analysis, as indicated in Figure 1D. Whole genome sequencing of the six mEp_immI_ phages was performed, and tBLASTx analysis revealed a conserved genome organization among these phages and that their proteins share high similarity rates (>80%, Figure 2A). Annotation of the sequenced genomes and mapping of the structural protein-coding genes (Figure 1D, top) indicated the presence of clusters of genes with related functions (Figure 2A).

### 3.2 Comparative phylogenomics analyses of mEp_immI_ phages

The genome of the archetype mEp021 phage (accession number MH706966.1), which was deposited in GenBank in 2018, was used to retrieve homologous phages and prophages in the GenBank (NCBI) database using BLASTn, with the parameters described in the Methods section. This allowed the identification of 42 additional viral complete genomes (Table 1), which displayed homogeneous GC content (average 46 ± 0.003%), a conserved genome architecture and high similarity rates in protein sequence (Supplementary Figure S3). Hence, these viral genomes were included in subsequent analyses as members of the mEp_immI_ group. Whole-genome phylogenetic analysis indicated that all mEp_immI_ phages and prophages (*n* = 48) constitute part of the same evolutionary branch, that bifurcates into two main subgroups (Figure 2B); several lambdoid and non-lambdoid phages were included as an external *Caudoviricetes* reference group. The segregation pattern of the six mEp_immI_ phages sequenced in this work indicated evolutionary closeness among them (Figure 2B). Intergenomic distance calculations using VIRIDIC, confirmed this observation, and indicated the presence of 9 genera and 45 species among the 48 genomes analyzed (Figure 2C), according to current criteria for phage taxonomy (Turner et al., 2024).

**Figure 3:**
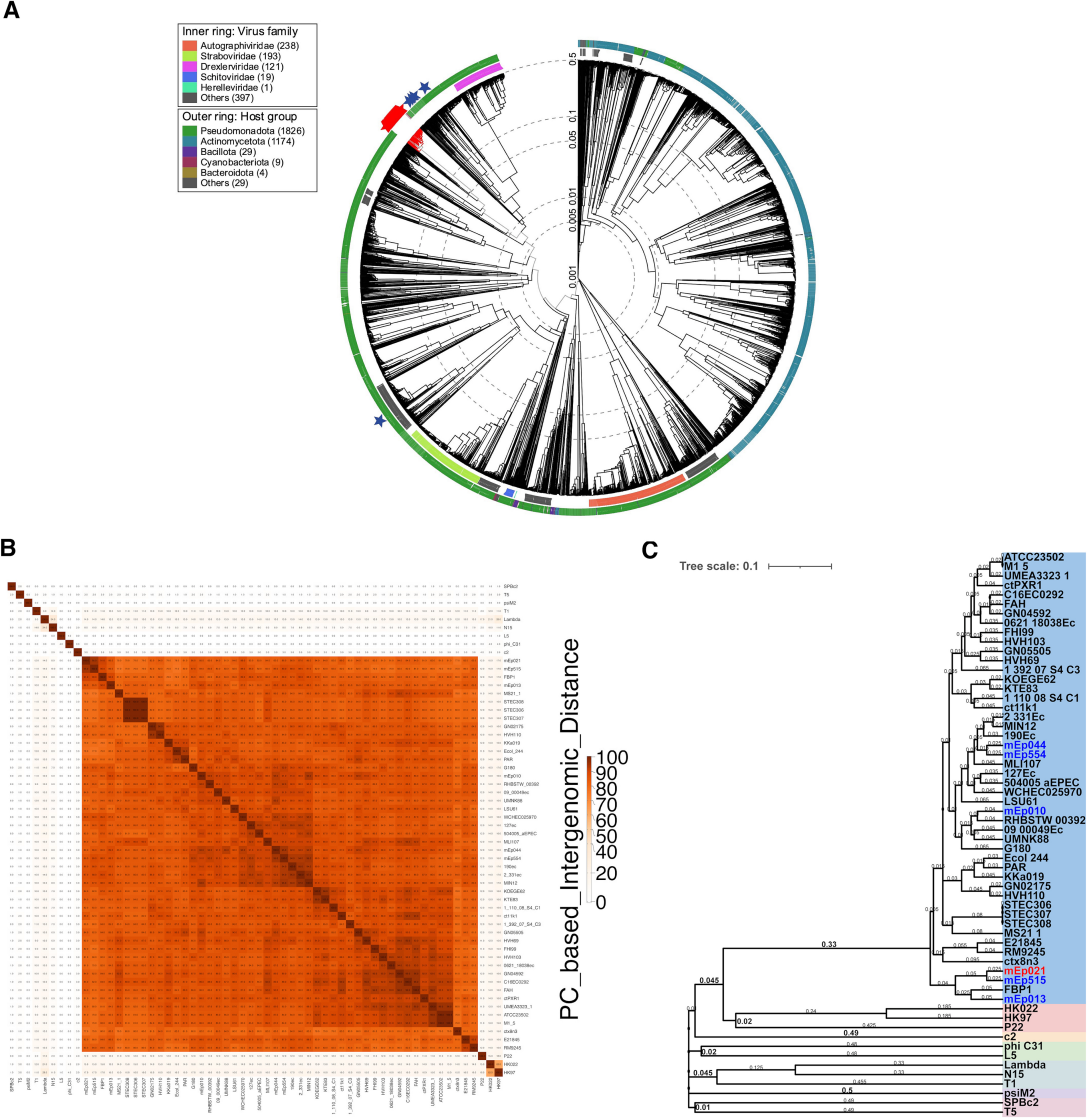
**(A)** Proteomic tree analysis (i.e., a dendrogram representing proteome-wide similarity relationships computed by tBLASTx) including the 48 mEp_immI_ phages and >5,600 dsDNA phages from the ViPTree 4.0 database. The mEp_immI_ phages/prophages are clustered in one separate branch (red marks); reference phages λ, T5, N15, HK022 and HK97 were included (blue stars). **(B)** Heatmap representing the pairwise intergenomic distances calculated from the conserved protein clusters (PC) using VirClust; the 48 mEp_immI_ genomes as well as 12 external reference phage genomes were included. Darker orange tones represent higher similarity values. **(C)** VirClust hierarchical clustering of phage genomes based on their PC-calculated intergenomic distances. The tree corresponding to bootstrap probability values is shown. The 48 mEp_immI_ genomes (blue background) segregate apart from the reference phage set (colored background). The six representative phages that were experimentally characterized in this work are indicated in blue and red font.

Further, tBLASTx analysis integrating the available proteomes from >5,600 dsDNA reference phages deposited in the ViPTree server, revealed that the mEp_immI_ group constitutes a compact and independent branch, and further separation from the reference group was observed when the 42 homologous phages and prophages were incorporated into the analyses (Figure 3A and Supplementary Figures S4A,B). These results demonstrate that the mEp_immI_ phages constitute a novel group, distinct to previously described bacteriophage clades. Intergenomic distance calculations based on conserved protein clusters using VirClust (Supplementary Figures S4C,D), confirmed that the 48 mEp_immI_ genomes belong to a separate branch, when compared against 62 phage genomes corresponding to the 12 external reference phages and 50 neighboring phage genomes observed in the ViPTree analysis (Supplementary Tables S2, S3 and Supplementary Figure S4A); this analysis indicated that the mEp_immI_ phages correspond to a unique viral genome cluster (VGC 1, Supplementary Table S5), while reference phages were coherently clustered, such as HK022 and HK97 (VGC 7, subfamily Hendrixvirinae), P22 (VGC 12, genus Lederbergvirus). At this distance threshold, phage λ (genus Lambdavirus) is clustered together with phage N15 (genus Ravinvirus), however, at 0.65 distance threshold these phages are properly segregated to distinct VGC, while the VGCs corresponding to the aforementioned reference phages and the mEp_immI_ group maintain their composition (data not shown).

**Figure 4:**
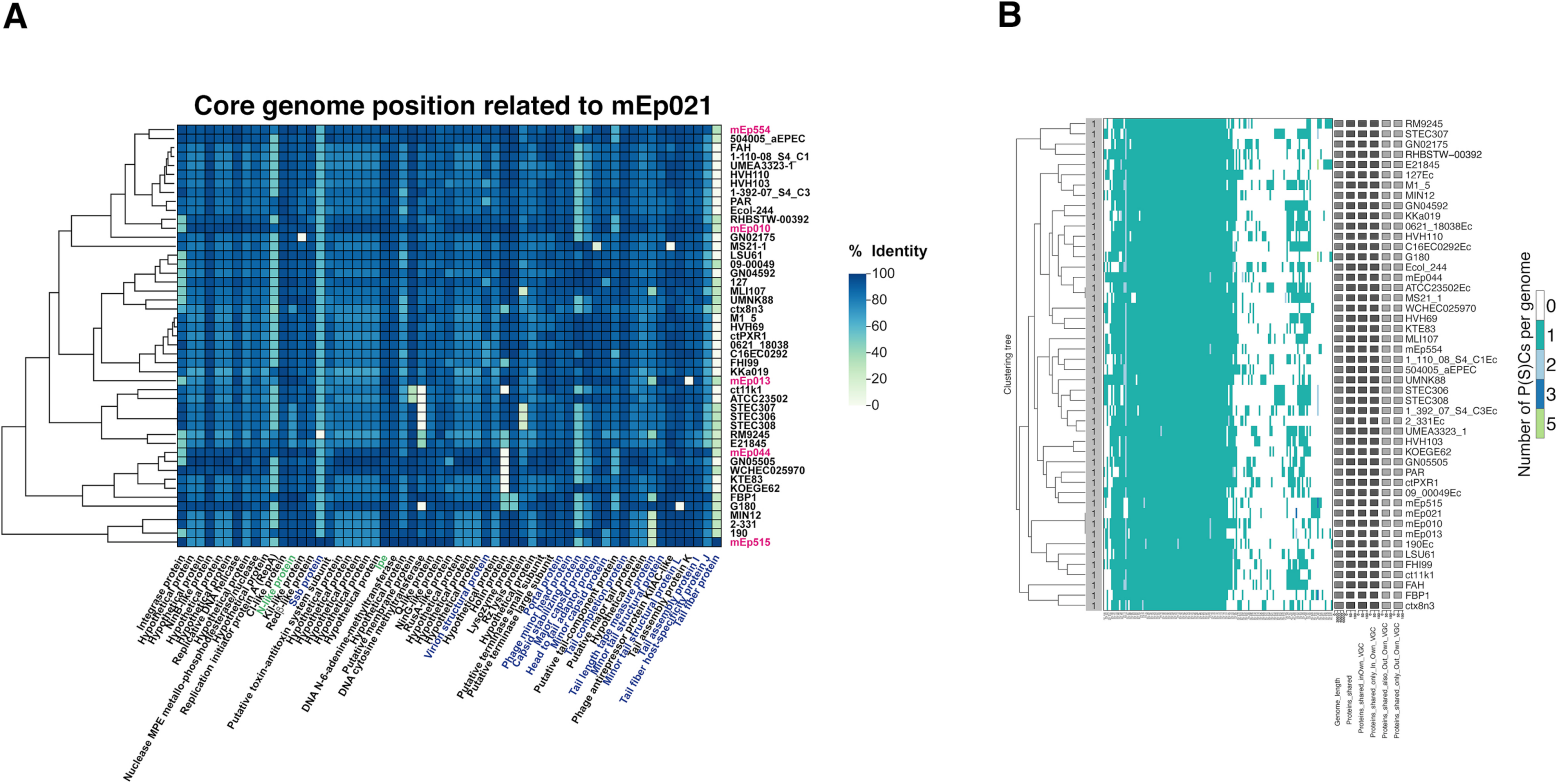
**(A)** Heatmap of the 59 core proteins manually identified from the 48 mEp_immI_ phage genomes. Taking the proteins of mEp021 as reference, the sequence conservation of each protein is depicted in a color gradient, where dark blue tone represents 100% identity, and light green tones indicate similarity decrease toward 0%. The ordering of proteins (columns) is fixed and is displayed according to the relative genomic position of their respective coding genes; the function of each protein is annotated at the bottom of the figure. Clustering of phages and prophages (rows) correlates with the overall protein similarity values. The mEp021 proteins identified by MS in this work are indicated in blue font, previously characterized proteins are in green font, and the six mEp_immI_ phages from our collection are denoted with pink font. **(B)** Automated VirClust analysis on the 48 mEp_immI_ phage genomes revealed 56 core proteins. From the left portion of the figure to the right: dendrogram of phage genomes according to protein cluster (PC)-calculated intergenomic distances (using a 0.9 distance threshold), viral genome cluster (VGC) number, PC distribution on the viral genomes, viral genome statistics and genome identifier.

The 48 mEp_immI_ genomes showed high synteny, containing from 106 to 128 genes according to our gene calling procedure (see Methods). Taking into account the relative position of each gene in the 48 phage genomes, the identity of all the predicted proteins was manually assessed in order to determine the core, flexible and unique genes in this group. We used the mEp021 genome as a reference, as the virion structural proteins were consistently mapped by MS analysis. This analysis revealed that 47.16% of the total genes (2813/5965) corresponded to core genes, representing 59 distinct gene products that were shared among all the analyzed genomes (Figure 4A). The gene products that were coded in several genomes but not all, were identified as flexible (49.14%; 2,931/5,965 genes), and were mainly located in the variable regions of these phages. Finally, 3.7% (221/5,965) corresponded to unique gene products. Automated analysis using the VirClust tool yielded from 87 to 103 called genes per genome, and indicated that the core genome included 57.4% of the total genes (2649/4619), corresponding to 56 core proteins (Figure 4B); 42.1% (1946/4619) were accessory genes and 0.5% (24/4619) were unique gene products. It should be noted that the core gene sets from the manual and automated analyses shared 50 common gene products (84.7 and 89.2% coincident genes, respectively), which were further considered as the confirmed core genome. The genes that were not common to both analyses mostly corresponded to hypothetical proteins.

### 3.3 The mEp_immI_ phages use a CI_λ_-like repressor protein

**Figure 5:**
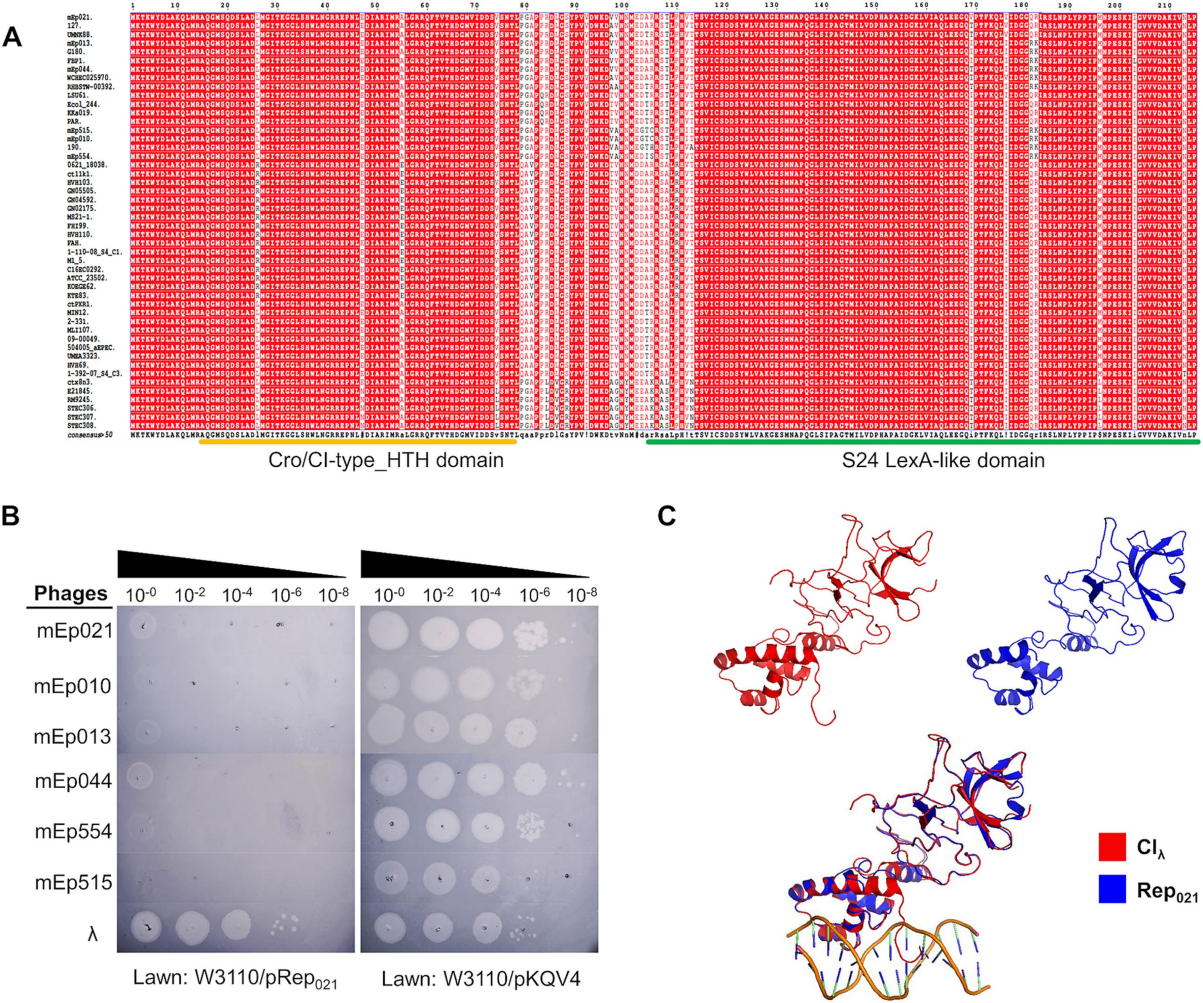
The mEp021 repressor protein is unique and is highly conserved in the mEp_immI_ group. **(A)** Alignment of the 48 mEp phages/prophages repressor proteins, showing high conservation among all the group. **(B)** Phage exclusion assays were performed in the transformed W3110 host strain expressing the repressor Rep_021_ from phage mEp021. Infection of phages mEp021, mEp010, mEp013, mEp044, mEp554, and mEp515 was inhibited; conversely, λ phage proliferated as expected. **(C)** The Rep_021_ repressor protein (blue) was modeled using the Phyre^2^ server, and the resulting structure was superposed with the CI_λ_ (red) crystallographic model (accession number 3BDN); the functional domains retain high structural similarity.

Lysogen immunity is regulated by the phage repressor protein, and constitutes one of the primary characteristics that allowed the identification of mEp_immI_ phage group (Kameyama et al., 1999; Stayrook et al., 2008; Thomason et al., 2019; Sedhom and Solomon, 2023). Alignment of the predicted repressor proteins of these phages indicated a high sequence similarity among them (95.95%), displaying conserved Cro/CI-type HTH and S24 LexA-like domains, according to InterProScan analysis (Figure 5A); these domains are required for DNA binding and protein dimerization, respectively. In order to demonstrate its functionality, the coding sequence of a representative repressor protein, Rep_021_ of phage mEp021, was inserted in a low copy-number expression vector; as expected, expression of Rep_021_ in a transformed W3110 strain blocked the infection of other mEp_immI_ phages (Figure 5B). Moreover, superposition of the Rep_021_ computational protein model and the CI_λ_ repressor crystallographic model (Figure 5C and Supplementary Figure S6B) revealed a remarkable structural similarity, especially in the regions corresponding to both functional domains of these proteins, despite that the amino acid sequences of the Cro/CI-type HTH domain at the amino-terminal region are less conserved.

### 3.4 mEp_immI_ phages and prophages have four types of integrases

**Figure 6:**
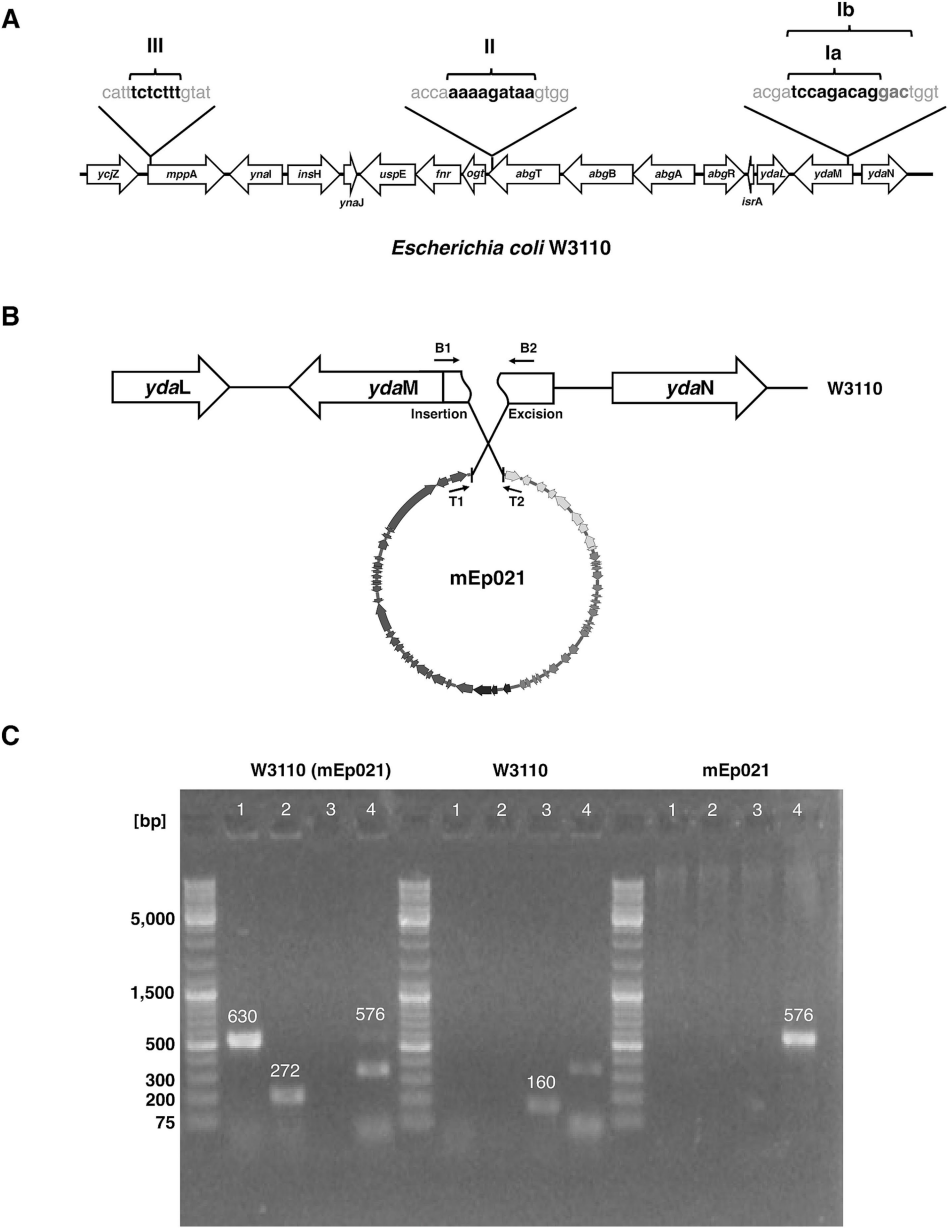
Insertion sites of mEp021 and mEp_immI_ prophages in the host genome. **(A)** *E. coli* genes are indicated in white arrows outlined in black, and the genomic positions of the prophage insertion sites are indicated with black lines; integration sequence motifs are shown. The associated integrase protein type is indicated above each integration site, respectively. **(B)** Schematic representation of the experimental strategy to validate the insertion site of the mEp021 bacteriophage in the W3110 strain. PCR primers (black arrows) were designed to amplify from the *att* site at the left and right ends of the mEp021 DNA (T1 and T2), and from the predicted insertion sites within the *yda*M host gene (B1 and B2). W3110 genes are represented by black arrows; the integration site disrupts the *yda*M gene (segmented arrow). The mEp021 genome is shown in circular form at the bottom. Diverse primer combinations were tested, and the predicted product sizes are indicated. **(C)** PCR validations of mEp021 integration site in its W3110 lysogen. The W3110 (mEp021) lysogen, W3110 and mEp021 DNA templates were evaluated, as indicated in the top of the gel images. The numbers 1, 2, 3 and 4 indicated on the top of the gels correspond to the primer combinations denoted in **(B)**. For W3110 (mEp021) template: lane 1, amplification of the right attachment site yielded the expected 630 bp amplicon (primers T1/B2); lane 2, amplification of the left attachment site (272 bp amplicon, primers T2/B1); lanes 3, no amplification; lane 4, a faint 576 bp band corresponding to episomal mEp021 was detected, as well as one minor unspecific amplicon. For W3110 template: only the 160 pb product corresponding to the *yda*M region amplification was observed (lane 3, primers B1/B2). For mEp021 template: only the 576 bp amplicon corresponding to the integration region of the mEp021 genome was observed (lane 4, primers T1/T2); the mEp021 DNA was obtained from CsCl-gradient virion purification to avoid bacterial DNA contamination.

Once the phage has ejected its DNA into the host cell, it may undergo lysogeny. During this process, the phage DNA may integrate into the host genome, using an integrase protein which is generally phage-encoded and is located nearby to the insertion sequence motifs. These phage insertion sequence motifs are similar to the host insertion sites and may correspond to distinct loci in the bacterial genome; these motifs are specifically recognized by the integrase proteins. Hence, we mapped the insertion site in the host genome and identified the insertion sequence motifs of each phage, as well as the integrase gene they, respectively, harbored. For this, we analyzed the host sequences located upstream and downstream to the 38 homologous mEp_immI_ prophage genomes that were found integrated in *E. coli* genomes from GenBank, and the W3110 (mEp021) lysogen was included (Supplementary Figure S7A). Different *att* sites and motifs were identified. For most phages, the integration site was located within the 5′-terminal region of the *yda*M gene, while for other phages the integration site was found at the 3′-terminal region of the *abg*T gene, or at the initiation region of the *mpp*A gene (Figure 6A and Table 2).

**Table 2:** Distribution of phages and prophages according to the identified integrase groups.

mEp_immI_ integrases group				
Ia	Ib	II	III	IV
*ct11K1	*0621–18038	*mEp010	LSU61	**ctx8n3**
*ctPXR1	*mEp515	*mEp044		
1–110-08_S4_C1	*mEp554	*mEp013		
ATCC23502	mEp021	09–0049		
Ecol_244	1–392-07_S4_C3	UMNK88		
FHI99	504005_aEPEC	RM9245		
HVH110	MIN12	RHBSTW-00392		
HVH69	2–331	GNO5505		
HVH103	FBP1	GNO4592		
KOEGE62	G180	E21845		
KTE83	STEC306	190		
MS21-1	STEC307	127		
C16EC0292	STEC308	▷ MLI107		
FAH	WCHEC025970			
GN02175				
KKa019				
M1/5				
PAR				
UMEA3323-1				

*The host insertion site was inferred from the viral integrase homology analysis.

▷ Insertion site with ~ 7000 bp deletion.

Phages are in bold and prophages in black font.

The mEp021 archetype phage for the mEp_immI_ group is indicated in red font.

We identified genes coding for integrase proteins in all the mEp_immI_ phages. All of these corresponded to the tyrosine-recombinase family (Groth and Calos, 2004) and amino acid sequence alignments allowed the recognition of four main types or clusters of these enzymes: types I (a and b), II, III and IV (Supplementary Figure S7B). We hypothesized that the use of different attachment sites would be related to the different integrase sequence-types. The results were consistent with such hypothesis, since the phages displaying the same attachment sites harbored the same integrase sequence-types: phages integrating at the *yda*M locus code for integrases types Ia and Ib; those integrating at *abg*T code for type II integrases, and the ones integrating at *mpp*A bear type III integrases (Table 2). Further, a unique integrase was found in the genome of phage ctx8n3 (integrase IV), a phage identified from metagenome sequences (Table 1); however, its host integration site could not be determined because the sequence upstream to the phage integrase (i.e., phage genome 5′ terminus) did not match the W3110 reference strain genome, and no sequence downstream to the phage genome was available.

As revealed by this analysis, phages mEp010, mEp013, and mEp044 harbor type II integrases, while phages mEp021, mEp515 and mEp554 code for type Ia integrases. Hence, the host integration site of archetype mEp021 phage was experimentally verified by colony PCR assays in a fresh W3110 lysogen, using primers flanking the *att*R (T1 and B2) and *att*L (T2 y B1) sites (Figure 6B), and the results confirmed the predicted attachment sites (Figure 6C).

### 3.5 The mEp_immI_ phages contain an N_λ_-like early antitermination system

**Figure 7:**
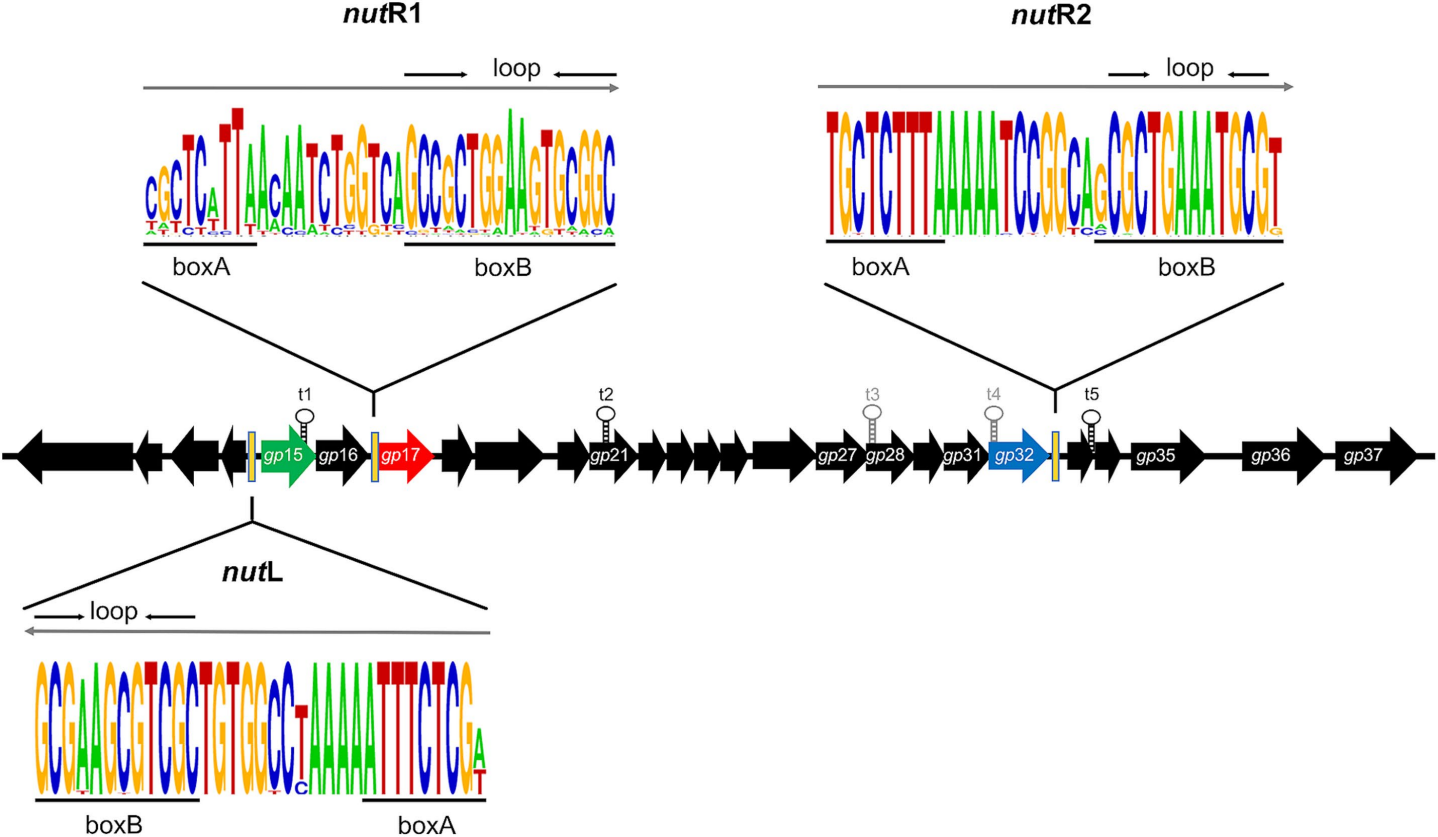
The antitermination system is highly conserved among the mEp phage group. Consensus sequences of the three *nut* sites (containing the boxA and boxB motifs, respectively) among the mEp_immI_ group is represented in the WebLogo format (https://weblogo.berkeley.edu/logo.cgi) (Crooks et al., 2004). The direction of transcription is indicated with gray arrows. The positions of these *nut* sites in the mEp021 genome are indicated by black bars; the *nut*L site is located upstream of the *gp*15 gene (coding the Rep_021_ repressor, represented as green arrow); the *nut*R1 site is upstream of the *gp*17 gene (N_λ_-type antiterminator, represented by a red arrow), and the *nut*R2 site is downstream of the *gp*32 gene (a putative N-cytosine-methyl-transferase, represented by a blue arrow). The predicted (t3 and t4) and experimentally demonstrated transcriptional terminators (t1, t2, and t5) are indicated in gray and black font, respectively.

It has been shown that the N_λ_-like protein Gp17 is responsible for the early antitermination process observed in phage mEp021 (Valencia-Toxqui et al., 2023). Its coding gene *gp*17 was present in all the studied mEp_immI_ genomes, and the arginine residues at positions 17, 19, 20 and 24 were fully conserved, indicating that this domain is fundamental to protein function (Supplementary Figure S8); arginine in position 21 was conserved to a lesser extent (45 out of 48 phages). Gp17 acts upon three N*ut* sites (*nut*R1, *nut*R2, and *nut*L), which are located in a similar genomic position among all the mEp_immI_ phages. The *nut*R2 and *nut*L sites show less sequence variability than the *nut*R1 (Figure 7), however, the adenine-rich region of the boxB recognition loop was well conserved in all three N*ut* sites, suggesting that the early antitermination process is similar among all the mEp_immI_ phage group.

### 3.6 Bacterial outer membrane proteins involved in the infection of mEp_immI_ phages

**Figure 8:**
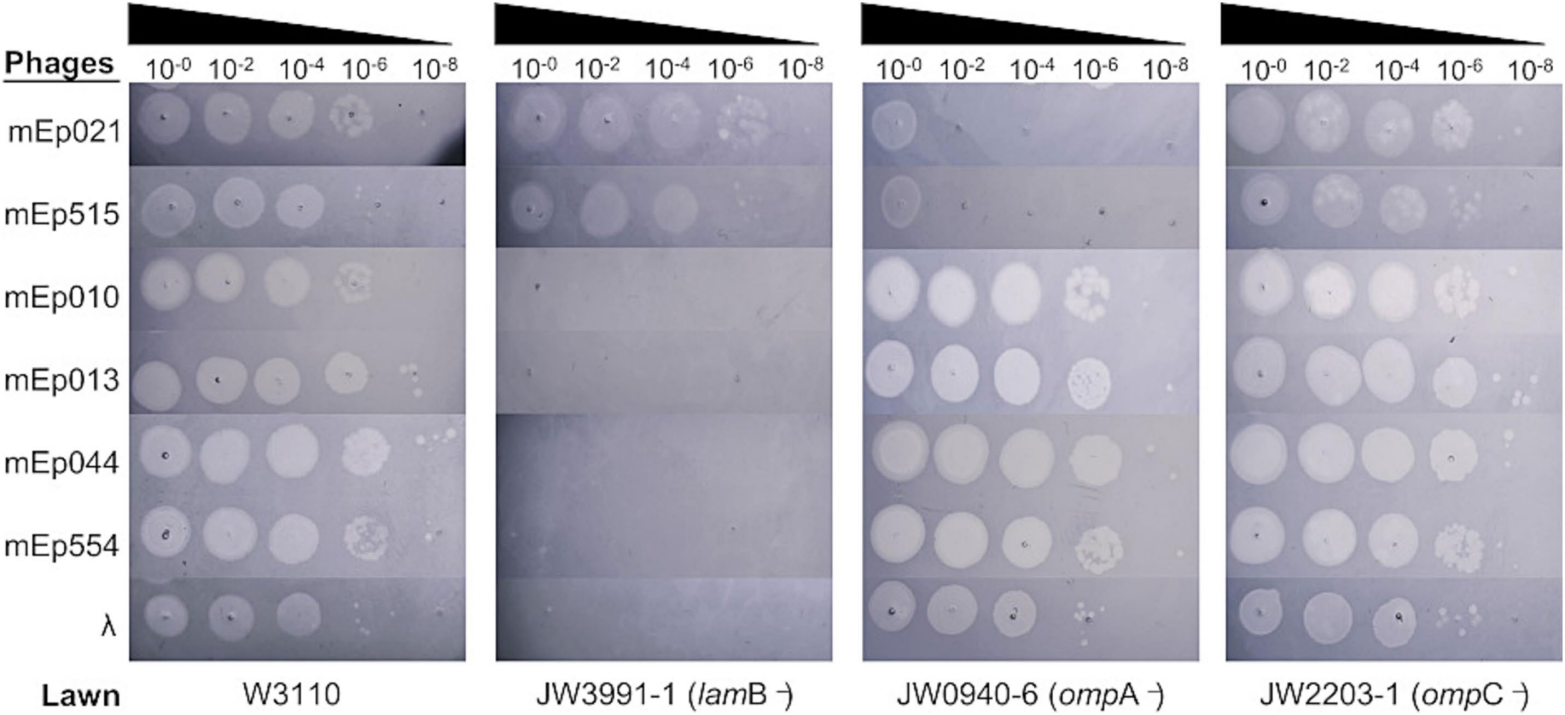
Identification of the main host (OMR) used by the 6 representative mEp_immI_ phages. Infection assays were performed on wild-type strain W3110 and three different Keio mutants (*omp*A^−^, *lam*B^−^, and *omp*C^−^), as labeled. Serial dilutions of bacteriophages were spotted onto bacterial lawns, as indicated. According to the observed lysis spots, the OMR OmpA is required for infection of phages mEp021 and mEp515, while the OMR LamB is required for mEp010, mEp013, mEp044, and mEp554.

One of the fundamental steps for phage multiplication is the infection of their hosts. For this, different OMR proteins of the bacterial cell may be recognized by the phage particles, in order to adsorb and eject their genome into the host. Hence, we determined the host OMR proteins used by our six mEp_immI_ phages and analyzed the phage structural protein that is involved in such recognition. For this, we performed a series of infection assays using several outer membrane protein null-mutant strains of the Keio collection (Datsenko and Wanner, 2000; Baba et al., 2006), which are knockouts for different non-essential genes of *E. coli*. As observed in Figure 8, in the *lam*B knockout strain the infection of phages mEp010, mEp013, mEp044 and mEp554 was completely inhibited, while the *omp*A knockout strain displayed a strong inhibition of mEp021 and mEp515 infections (barely yielding ~10^2^ PFU/mL).

The recognition and binding to the bacterial OMR proteins is mediated by phage J proteins. A multiple alignment of the predicted J proteins of the mEp_immI_ phages revealed that, despite all these proteins display a different size, a notable sequence conservation is observed, mainly in the amino-terminal region (Supplementary Figure S10); this region also shares structural similarity with the J protein from phage λ (data not shown). The carboxy-terminal region shows more sequence variation, and contains repeats of different length (throughout positions 950–1,475), a conserved stretch (position 1,476–1,690), and a less conserved terminus. The latter is involved in physical contact with the bacterial receptor, as shown in phage λ studies (Ge and Wang, 2024; Wang et al., 2024).

### 3.7 The Lpp exclusion proteins of the mEp_immI_ phages

**Figure 9:**
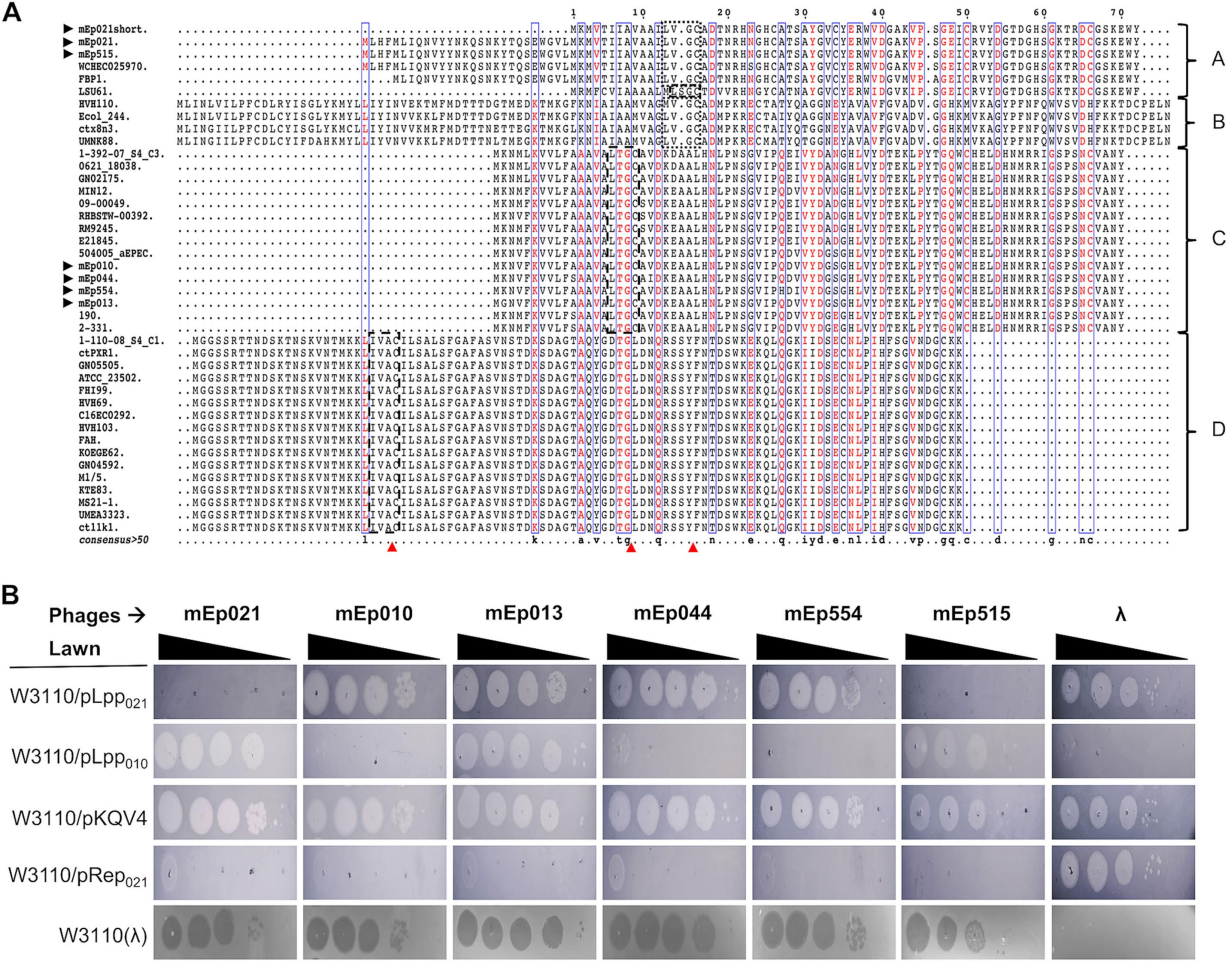
Lpp lipoproteins mediate mEp_immI_ phage exclusion. **(A)** Alignment of the Lpp predicted lipoprotein sequences of 40 phages and prophages. The lipo-box motifs are shown in black dashed boxes, the cleavage sites are indicated with red arrows; once processed, the protein starts with cysteine, and the subsequent residue determines the final location of the lipoprotein. **(B)** Infection assays using W3110 strains transformed with pLpp_021_ or pLpp_010_ plasmids that express the lipoproteins Lpp_021_ or Lpp_010_, respectively. Serial dilutions of phages were spotted onto distinct bacterial lawns, and the formation of lysis spots indicating phage development was evaluated. Expression of Lpp_021_ inhibited phages mEp021 and mEp515, while Lpp_010_ inhibited phages mEp010, mEp044, mEp554, and λ. None of the tested lipoproteins could inhibit the development of phage mEp013.

The superinfection-exclusion mechanism has been described in several lysogens and it can be mediated by lipoproteins, among other types of proteins. We searched the mEp_immI_ genomes for lipoprotein genes through the identification of the lipobox signature motif ([LVI][ASTVI][GAS][C]), which is recognized by the lipoprotein signal peptidase (Lsp) that cleaves the signal peptide and promotes further allocation of the lipoprotein on the bacterial membrane (LoVullo et al., 2015). Our results indicated that 40 out of the 48 mEp_immI_ phages contain at least one gene that putatively codes for a lipoprotein. A multiple alignment of the predicted lipoproteins allowed the identification of four sequence types (Figure 9A). Two of these types were represented in the phages sequenced in this work: type-A lipoprotein was found in mEp021 and mEp515, and the respective amino acid sequences were identical; we selected the coding gene from mEp021, as this is the mEp_immI_ archetype phage that has been best described so far. Type-C lipoproteins were found in mEp010, mEp013, mEp044 and mEp554, and the respective amino acid sequences were not identical: the mEp013 lipoprotein harbored a K > G substitution at position 2, the mEp044 an A > S substitution at position 11, the mEp554 lipoprotein displayed a Q > H substitution at position 38 and a conservative substitution V > I at position 40 (see Figure 9A). The consensus sequence of this alignment corresponded to that of the mEp010 lipoprotein, which contained the most conserved amino acids in each position in relation to the other lipoprotein sequences of this type. In addition, the mEp010 phage was the most distant to the archetype phage mEp021 in the ViPTree analysis (Supplementary Figure S4A), therefore we selected its lipoprotein coding gene as representative for the type-C lipoproteins. We cloned the aforementioned genes in an expression vector in order to assess their function through phage infection assays (Arguijo-Hernández et al., 2018), and the results showed that the mEp021 type-A lipoprotein (Lpp_021_) excluded phages mEp021 and mEp515, while the mEp010 type-C lipoprotein (Lpp_010_) was able to exclude phages mEp010, mEp044, mEp554 and λ (Figure 9B).

## 4 Discussion

Phages of the order *Caudoviricetes* (tailed virus) have been reported as the most frequent communities in both healthy individuals and inflammatory bowel syndrome patients; consistently, the morphotypes myovirus, podovirus and siphovirus were recognized as the most predominant entities in gut (Ansari et al., 2020; Coughlan et al., 2021). As such, the mEp_immI_ phages were found to be as prevalent as the lambdoid phages in clinical samples of human feces (Kameyama et al., 1999), and we also identified complete and incomplete homologous phage genomes in two metagenome projects (NCBI Bioprojects 285453 and 862966, registered in 2015 and 2022, respectively) (Tisza and Buck, 2021). We started the characterization of these phages using mEp021 as a representative of the group, due to its ability to induce a hemolytic phenotype in its *E. coli* MC4100 lysogen, which is mediated by the phage-encoded Ipe protein (Kameyama et al., 2001; Martínez-Peñafiel et al., 2012). In addition, mEp021 has an antitermination mechanism similar to that of λ, but with the use of three different *nut* sites instead of the typical two (Valencia-Toxqui et al., 2023).

Grouping of phages based on similarities in their genome organization and gene regulation can provide insights into their biological behavior (Valencia-Toxqui and Ramsey, 2024). In this work, we extended the characterization of the mEp_immI_ phages, including mEp021, mEp010, mEp013, mEp044, mEp515, and mEp554, to provide structural and functional data supporting the establishment of a new group of coliphages, following current taxonomical criteria and traditional biological characterization approaches. TEM analysis (Figure 1A) confirmed a common siphoviral morphotype in the analyzed coliphages, however, the differential host range reported (Kameyama et al., 1999) and the electrophoretic patterns observed both in the DNA restriction and structural protein SDS-PAGE analyses presented herein, indicated that each of these phages is a particular entity (Figures 1B,C). Whole genome sequencing confirmed this, and despite the fact that these phages display diverse characteristics (Kameyama et al., 1999, 2001), their overall sequence similarity is high and they share a common genome architecture (Figure 2A).

MS analysis of the mEp021 structural proteins allowed the mapping of the structural protein gene cluster in the phage genomes (Figure 1D, black arrows), including those coding for the phage tail fiber protein J, tail length tape measure protein, portal protein, tail tip protein, coat protein, among others. Interestingly, the coding genes of two structural proteins were distant to the main cluster, both being located upstream to the lysis region. One of such genes codes for a putative single-stranded DNA binding protein (Ssb); to our knowledge, this type of proteins does not form part of the viral structure but are rather involved in the stimulation of viral DNA replication, as it was previously shown for a Ssb protein from phage φ29 (Soengas et al., 1994). We therefore suggest that this protein may participate in DNA protection or organization. The other mislocated gene codes for a hypothetical virion structural protein of unknown function, which is conserved in all the analyzed genomes with a > 60% similarity. Distant homology searches using PLMSearch (Liu et al., 2024) indicated that this protein is similar to a translation repressor protein from *E. coli* phage RB69, which is an RNA-binding protein, and to the uncharacterized protein YorC from the SPbeta prophage (similarity scores = 0.9963, in both cases); a helix-turn-helix DNA binding motif was recognized in the first hit (Uniprot accession Q01751), suggesting that this phage protein may physically interact with DNA. As this protein was detected in the MS analysis as part of the viral structural proteins (which were obtained from phages purified through a CsCl-gradient), we suggest it is possibly involved in the conformation of viral particles.

A BLASTn search in the GenBank database using mEp021 as reference, allowed the identification of 42 additional homologous mEp_immI_ prophages and phages (Table 1), from *E. coli* genomes (*n* = 38) and metagenome sequences (*n* = 4). This set of viral genomes substantially differed from reference phage genomes of the order *Caudoviricetes*, as demonstrated by subsequent phylogenetic analysis, which indicated that these phages warrant a formal taxonomic classification. Several analysis tools such as VIRIDIC, ViPTree, and VirClust have shown to be consistent with the criteria set by the Bacterial Virus Subcommittee of the International Committee on Taxonomy of Viruses (ICTV) to assign taxonomic ranks at different levels (Turner et al., 2021, 2024). Despite their widely diverse geographical origins, an elementary alignment analysis using whole genome sequences, showed that the mEp_immI_ phages constitute a well delineated group that clusters apart from reference siphovirus (Figure 2B); this was confirmed by intergenomic distance analyses using VIRIDIC (Figure 2C). These results allowed the identification of 8 genera and 45 species among the 48 genomes examined, according to the demarcation criteria of 95% sequence similarity for species and 70% for genus in whole-genome analyses (Turner et al., 2024). Further, whole-proteome comparative analysis showed that the 48 mEp_immI_ phages form a cohesive and monophyletic group, sharing a significant proportion of orthologous genes (Figure 3A and Supplementary Figure S4A,B), when compared to >5,600 dsDNA phage proteomes; this is consistent with a family-level classification (Turner et al., 2021, 2024). These results were further supported by the analysis using VirClust, which calculates intergenomic distances based on the identified protein clusters, confirming the segregation of the mEp_immI_ group as an independent branch, in both analyses using the dataset of 12 reference phages (Figures 3B,C) and a larger dataset including the neighboring viral genomes observed in the ViPTree analysis (*n* = 110) (Supplementary Figures S4B,C). VirClust analysis confirmed that the mEp_immI_ group constituted a single viral genome cluster (VGC) (Supplementary Table S5). Additional analyses will be required to determine whether viral sub-families are present among this phage group.

All the mEp_immI_ phages displayed high sequence similarity and genome synteny, as revealed by the tBLASTx analysis (Supplementary Figure S3). This indicates that these phages may share numerous biological similarities and, considering their common ecological niche, it is possible that these phages course an early phase of evolutionary diversification, as previously suggested (Kameyama et al., 1999). Their genes are clustered according to related functions, and this includes the genes involved in replication/recombination, the lysis-lysogeny regulation, packaging, lysis and viral morphology gene clusters (Figure 2A). However, sequence and gene content variation was also observed, involving genes such as integrases, those involved in lysis/lysogeny regulation, the J protein and the tail tip protein genes, among others. Considering this, we analyzed the whole gene content and its conservation among all these genomes. A manual pangenome analysis indicated that 47.16% of the total number of identified genes are present in all the 48 genomes analyzed, and these correspond to 59 core gene products that are related to all the functional clusters (Figure 4A). Variable or accessory genes constituted 49.14% of the total genes and were mainly located in the regulation cluster; we suggest that this region is the main contributor to group diversification. In addition, an automated analysis using VirClust indicated the presence of 56 core genes (Figure 4B). The majority of the genes that were not coincident between these analyses (9 in the manual analysis and 6 in VirClust, respectively) corresponded to hypothetical proteins. From these, 4 of the proteins identified in the manual analysis are probably related to phage functions, including a Kil-like protein, DNA cytosine methyltransferase, phage antirepressor protein KilAC-like, and a phage tail assembly protein K. Conversely, two proteins were recognized among the non-coincident proteins from the VirClust analysis, including an AlpA-family transcriptional regulator from bacteria and phages, and a centrosome-associated protein, which is a heterologous finding but may refer to a nucleic acid binding protein as well. Taking these results into account, we confirmed the presence of at least 50 core genes in the studied mEp_immI_ genomes, which were coincident between the manual and automated analyses.

In order to validate relevant genes from the functional clusters, we performed a series of experiments using the six sequenced mEp_immI_ phages from our collection. A prime method in phage characterization is to evaluate the auto-exclusion mechanism of a phage from its own lysogenic strain, as well as the exclusion of phages from the same group or family (Dulbecco, 1952). The 50 original phages from the immunity I group were partly characterized using this approach (Kameyama et al., 1999), which implies that there is similarity in their main repressor proteins. Consistently, we found high similarity among the sequences of the repressor proteins of the 48 mEp_immI_ phages in this study, showing the two highly conserved functional domains: Cro/CI-type HTH and S24 LexA-like (Figure 5A and Supplementary Figure S6), which are typical features of CI-type repressors. We demonstrated Rep_021_ exclusion activity using transformed strains expressing this protein, which inhibited mEp021, mEp010, mEp013, mEp044, mEp515 and mEp554 infections (Figure 5B). Noteworthy, the use of highly concentrated phage preparations produced a growth inhibition effect, but no isolated lysis plaques were observed. This could be result of a non-specific lysis due to lysis from without (Abedon, 2011). Notable amino acid sequence differences were observed between the CI_λ_ repressor and the 48 Rep predicted proteins (Supplementary Figure S5A), especially regarding the Cro/CI-type HTH domain. However, the three-dimensional structure of both functional domains is highly conserved (Figures 5C and Supplementary Figure S6B), according to the structural models of Rep_021_ obtained from three different prediction tools (see Materials and Methods). Despite the high structural similarity at the domain-level, the global conformation of Rep_021_ and CI_λ_ differs, probably due to the positioning of both domains, which is mediated by a hinge region in between. All these structural differences may account for specific biological properties. For instance, sequence variation in the HTH domains of CI_λ_ and Rep021 may lead to the recognition of different operator sequences; the six initial amino acid residues of CI_λ_ play a critical role in the recognition of the operator site, where Lys3, Lys4, and Lys5 were shown to be involved in direct physical interaction with the DNA bases (Clarke et al., 1991); a shorter N-terminal arm with two highly conserved lysine residues is observed in Rep021 (Supplementary Figures S6A,B), thus it is probably involved in a similar function. Moreover, it is possible that the self-cleavage activity of Rep_021_ is different to that of CI_λ_, since the C-terminal region contains the catalytic site for self-cleavage but lacks the specific amino acids of the cleavage site at the hinge region (A111-G112) (Sauer et al., 1982; Supplementary Figure S6A). This may explain previous observations in which UV radiation fails to induce mEp_immI_ lysogens to the lytic cycle (Kameyama et al., 1999).

The mEp_immI_ group harbors a post-transcriptional regulation system similar to the λ antitermination system, which requires host Nus factors as well as the phage N_λ_-like and Q_λ_-like proteins, to allow the development of these phages. In previous work, we experimentally characterized the N_λ_-like protein of mEp021 phage, namely Gp17, which is responsible for the early antitermination process in this phage. Unlike N_λ_, Gp17 acts upon 3 *nut* sites (*nut*R1, *nut*R2 and *nut*L) (Valencia-Toxqui et al., 2023). In this work, we show that Gp17 is highly similar among the mEp_immI_ phages, and conserves the ARM involved in the interaction with the boxB region of the *nut* site, which is determinant for its binding to RNA (Supplementary Figure S8). Consistently, the *nut* sites are also conserved in these phages (Figure 7), especially regarding the adenine-rich region in the boxB motifs. *nut*R2 and *nut*L are the most similar, while *nut*R1 displays higher sequence variability although it retains the boxA and boxB binding motifs. These results indicate that the mEp_immI_ phages harbor a similar antitermination system, that differs to that of λ due to the requirement of an additional *nut* site.

Despite the observed similarities in sequence, genome organization, repressor-related immunity control and antitermination, the mEp_immI_ phages displayed several biological differences among them. One of such differences involved the host genome integration sites, which were analyzed and compared between the 38 prophage genomes (corresponding to *E. coli* lysogen and metagenome sequences). We identified attachment sites within four distinct bacterial loci: *yda*M (motifs Ia and Ib), *abg*T (motif II), *mpp*A (motif III) and one which could not be mapped (motif IV), due to insufficient metagenome sequence data; each of these was separated by approximately 7 kb to the others. Most of the analyzed prophages were found inserted into the *yda*M locus, which codes for a putative diguanylate cyclase enzyme related to the expression of the biofilm-associated curli fimbriae (Weber et al., 2006; Figure 6A and Table 2). We assume that this represents the ancestral insertion site, and that subsequent variation has allowed the recognition of distinct insertion sites, which were less prevalent (Supplementary Figure S7A). For instance, the difference between sites Ia and Ib, is the presence of 3 additional base-pairs in the *att* motif of the latter (Figure 6A). The archetype phage mEp021 insertion site, corresponding to the Ib motif, was experimentally confirmed by PCR in W3110 (mEp021) lysogens (Figures 6B,C), but further experimental approaches will be required to determine whether the 3 additional base-pairs are essential for phage integration. Noteworthy, these integration sites differed from those reported for phage λ, involving a 16 kb stretch between the *gal* and *bio* loci (Landy and Ross, 1977). We refined the λ insertion sites through a similar lysogen analysis (*n* = 15; data not shown), revealing that this phage integrates in between the *ybh*C and *ybh*A loci, unlike the mEp_immI_ phages. Moreover, we identified four types of integrases in the mEp_immI_ genomes, according to the sequence alignments of predicted proteins (Supplementary Figure S7B). Interestingly, each of the four sequence-types of integrases specifically corresponded to an integration site observed in the lysogens, therefore the observed amino acid sequence variation could explain their specificities. Amino acid sequence analysis of the integrases revealed that the N-terminal region containing the DNA-binding domain was conserved, while the central and C-terminal regions harboring the catalytic domain displayed the most variability, which may account for the differential recognition and insertion to the diverse target regions. It is noteworthy that the insertion region recognized by prophage MLI107 displays a > 7 kb deletion, despite its integrase displays no relevant amino acid sequence differences when compared to group II integrases; therefore, this may be the result of a bacterial recombination event in the target region.

This novel group of phages also require different OMR proteins for infection (Figure 8). Similar to λ, the mEp010, mEp013, mEp044, and mEp554 phages use the bacterial LamB maltoporin, despite being non-lambdoid phages. Interestingly, the mEp021 and mEp515 phages require the bacterial OmpA OMR protein; few phages are known to use this receptor protein, mainly belonging to the T-even group (Schwarz et al., 1983). Noteworthy, the absence of OmpA does not completely inhibit the infection of these phages, suggesting that this protein acts as the main OMR, but alternative receptor proteins may also be used. These findings prove the diversity within the mEp_immI_ phages and reveal discrete biological similarities with heterologous phages. Sequence analyses of the predicted phage J proteins showed that the C-terminal region, which harbors the Receptor Binding Domain (RBD) (Wang et al., 2024), is variable (Supplementary Figure S10). Phylogenetic clusters based on the J protein alignments were consistent with the OMR groups observed and the presence of additional clusters suggests the possibility that even other receptor proteins, different to the ones characterized here, may be used by the remaining phages of the mEp_immI_ group (Supplementary Figure S11). This is analogous to what has been observed in the lambdoid group of the mEp collection, in which the use of FhuA rather than the LamB receptor is predominant, and OmpC is secondarily used (Hernández-Sánchez et al., 2008).

Finally, another differential characteristic among the mEp_immI_ phages is the superinfection-exclusion system, which can be mediated by lipoproteins that interfere with DNA ejection into the cytoplasm or block the host receptor protein. This system was present in 83% of the studied genomes and at least three distinct lipoprotein types were identified, according to amino acid sequence alignments (Figure 9A). These lipoprotein genes were located within the structural protein gene cluster of these phages and prophages. We experimentally tested the Lpp_021_ lipoprotein, corresponding to phages mEp021 and mEp515, belonging to the type-A lipoprotein present in phages which also use the OmpA OMR protein for infection; the amino acid sequences of these lipoproteins were identical. Consistently, expression of Lpp_021_ excluded phages mEp021 and mEp515. Likewise, expression of the type-C ancestral lipoprotein Lpp_010_ excluded almost all of the corresponding group phages which use the LamB receptor: mEp010, mEp044, mEp554 and, unexpectedly, phage λ. Despite the latter uses the same OMR, to our current knowledge this is the first instance in which expression of a non-lambdoid lipoprotein promotes phage λ exclusion, besides that there are no lipoproteins from lambdoid phages reported to exclude λ (Figure 9B). Remarkably, Lpp_010_ expression did not exclude phage mEp013 (also LamB-dependent), despite the amino acid sequence between the Lpp from mEp010 and the one from mEp013 differs in only one residue at position 2 (Figure 9A); this region belongs to the hydrophobic peptide leader that is processed by Lsp and is not expected to be involved in the exclusion mechanism. Thus, it is possible that the observed evasion of lipoprotein exclusion is mediated by the variation in the mEp013 protein that recognizes the LamB receptor, probably the J protein (see Supplementary Figure S10). However, with our current approaches, it is difficult to determine which domain of the J protein is involved in the physical recognition of the OMR.

In this work, we have characterized several fundamental aspects of the mEp_immI_ phages according to current taxonomic criteria, which include comparative genomics and proteomics analyses, and compiled evidence related to the most relevant biological traits of these phages, such as morphotype, phage infection, integration, immunity and lysogeny regulation, and super-infection exclusion (i.e., “lifestyle” and host factors) (Turner et al., 2024). According to whole genome alignments, intergenomic distance calculations and hierarchical clustering, these coliphages constitute a novel branch of *Caudoviricetes*, distinct from other known coliphages, representing 9 genera and 45 species. Whole proteome comparison against >5,600 dsDNA phages indicated that these phages form a separate branch with internal cohesion, consistent with a family-level classification. Traditional phage characterization often allows for the association of biological behaviors that may not be evident in taxonomic classifications, constituting a complementary approach that helps biologists understand the relationships and characteristics of specific phages or groups of phages. Therefore, we additionally presented a biological feature-based characterization of these phages, and the results revealed particular traits in which these phages differ from reference phages like λ, such as distinct genome integration sites or the use of an additional *nut* site for antitermination, but also revealed diversity within the group, as observed in the use of different OMR proteins or lipoproteins for infection and superinfection exclusion, respectively. Noteworthy, these phages were as prevalent as lambdoid phages in clinical samples of human feces and homologous phage genomes were identified from sources with diverse geographical locations, including metagenome samples. It is possible that this group has undergone minor diversification events, as indicated by the high similarity rates of their repressor proteins (95.95%), which account for the fact that these phages share the same immunity group. Contrastingly, lambdoid phages have developed widely diverse immunity groups (Kameyama et al., 1999, 2001). This conservation could also indicate that the emergence of the mEp_immI_ phages as *E. coli* parasites is recent, and that the original host could be another bacterial species. According to the ViPTree analysis, the branches closest to the mEp_immI_ cluster are mainly constituted by *Salmonella* and *Klebsiella* phages (Supplementary Figure S4A). In addition, we found two complete homologous prophage genomes in *Shigella flexneri* and *Shigella sonnei* strains (accession numbers: ABMBTU010000008 and AAZUZA010000004, respectively), displaying intergenomic similarities of 69.7 and 73.7%, respectively, according to the VIRIDIC analysis. Further exploration and genomic characterization of mEp_immI_ phages from different ecological niches will help to clarify the origins and evolution of this novel group of coliphages.

## Data Availability

The datasets presented in this study can be found in online repositories. The names of the repository/repositories and accession number(s) can be found in the article/Supplementary material.

## Footnotes

^1^ https://usegalaxy.org

^2^ http://www.bioinformatics.babraham.ac.uk/projects/fastqc/

^3^ https://www.bv-brc.org/

^4^ https://www.genome.jp/viptree/

^5^ http://multalin.toulouse.inra.fr/

^6^ https://www.ebi.ac.uk/jdispatcher/msa/clustalo

^7^ https://www.chiplot.online
