# Supplementary material for: Genomic and proteomic analyses of Nus-dependent non-lambdoid phages reveal a novel coliphage group prevalent in gut: mEpimmI
Source: Front Microbiol. 2025 Feb 24;16:1480411. doi: 10.3389/fmicb.2025.1480411 (PMC11893012; doi:10.3389/fmicb.2025.1480411)
Supplement: Supplementary file 2 [file Data_Sheet_2.pdf]

Table S1. Bacteria, bacteriophages and plasmids used in this study.

Strains bacteriophages and plasmids	Genotype/ Relevant markers	Sources or Reference
**Bacteria**		
W3110	F^-, λ^-, *rph*$^-$	Asakura Y., et al. (2009) ; Bachmann B.J. (1972)
JW0940-6 (*ompA*$^-$)	F^-, Δ(*araD-araB*)567, Δ*lacZ*4787(::*rrnB*-3), λ^-, Δ*ompA*772::kan, *rph*-1, Δ(*rhaD-rhaB*)568, *hsdR*514	Baba T. et al. (2006)
JW3996-1 (*lamB*$^-$)	F^-, Δ(*araD-araB*)567, Δ*lacZ*4787(::*rrnB*-3), λ^-, *rph*-1, Δ(*rhaD-rhaB*)568, Δ*lamB*732::kan, *hsdR*514	Baba T. et al. (2006)
JW2203-1 (*ompC*$^-$)	F^-, Δ(*araD-araB*)567, Δ*lacZ*4787(::*rrnB*-3), λ^-, Δ*ompC*768::kan, *rph*-1, Δ(*rhaD-rhaB*)568, *hsdR*514	Baba T. et al. (2006)
**Plasmids**		
pJET1.2	Transit vector, high copy number, AmpR	Lubys,A (2007)
pKQV4	Low copy number vector, AmpR	Strauch M.A. et al. (1989)
$pRep_{021}$	pKQV4 with *gp*15_{021} repressor gene from mEp021	This study
$pLpp_{021}$	pKQV4 with short lpp_{021} gene from mEp021	This study
$pLpp_{010}$	pKQV4 with lpp_{010} gene from mEp010	This study
**Bacteriophages**		
mEp021	Host Nus-dependent non-lambdoid bacteriophage	Kameyama L., et al. (1999)
mEp010	Host Nus-dependent non-lambdoid bacteriophage	Kameyama L., et al. (1999)
mEp013	Host Nus-dependent non-lambdoid bacteriophage	Kameyama L., et al. (1999)
mEp044	Host Nus-dependent non-lambdoid bacteriophage	Kameyama L., et al. (1999)
mEp515	Host Nus-dependent non-lambdoid bacteriophage	Kameyama L., et al. (1999)
mEp554	Host Nus-dependent non-lambdoid bacteriophage	Kameyama L., et al. (1999)
Lambda or W-1	Lambda wild type	CSH Collection

Table S2. Genome accession numbers of the 12 reference bacteriophages used in this study.

Reference bacteriophages	Accession number	Bacterial Host	Morphotype	Reference
λ	J02459	*Escherichia coli*	Siphovirus	https://ictv.global/report_9th/dsDNA/Siphoviridae
HK022	AF069308	*Escherichia coli*	Siphovirus	https://ictv.global/report_9th/dsDNA/Siphoviridae
HK97	AF069529	*Escherichia coli*	Siphovirus	https://ictv.global/report_9th/dsDNA/Siphoviridae
N15	AF064539	*Escherichia coli*	Siphovirus	https://ictv.global/report_9th/dsDNA/Siphoviridae
T1	AY216660	*Escherichia coli*	Siphovirus	https://ictv.global/report_9th/dsDNA/Siphoviridae
T5	AY543070	*Escherichia coli*	Siphovirus	https://ictv.global/report_9th/dsDNA/Siphoviridae
psiM2	AF065411	*Methanobacterium*	Siphovirus	https://ictv.global/report_9th/dsDNA/Siphoviridae
SPBc2	AF020713	*Bacillus*	Siphovirus	https://ictv.global/report_9th/dsDNA/Siphoviridae
c2	L48605	*Lactococcus*	Siphovirus	https://ictv.global/report_9th/dsDNA/Siphoviridae
phi-C31	AJ006589	*Streptomyces*	Siphovirus	https://ictv.global/report_9th/dsDNA/Siphoviridae
L5	Z18946	*Mycobacterium*	Siphovirus	https://ictv.global/report_9th/dsDNA/Siphoviridae
p22	NC_002371.2	*Salmonella typhimurium*	Podovirus	https://ictv.global/report_9th/dsDNA/Podoviridae

Table S3. Set of 50 heterologous bacteriophages that were close mEp_{immI} neighbors in the ViPTree analysis.

Accession Number	Host and phage name
NC_006949.1	Enterobacteria phage ES18
NC_010495.1	Salmonella phage Vi II-E1
NC_018279.1	Salmonella phage vB_SosS_Oslo
NC_019545.1	Salmonella phage SPN3UB
NC_019927.1	Cronobacter phage ENT47670
NC_021534.1	Vibrio phage pYD38-A
NC_031918.1	Salmonella phage 64795_sal3
NC_031924.1	Salmonella phage IME207
NC_042037.1	Aeromonas phage pIS4-A
NC_048197.1	Erwinia phage vB_EhrS_49
NC_048198.1	Erwinia phage vB_EhrS_59
NC_054636.1	Shigella phage Sf11 SMD-2017
NC_054637.1	Escherichia phage vB_EcoS Sa179lw
NC_054638.1	Salmonella phage vB_SenS_SB28
NC_054639.1	Salmonella phage Skate
NC_054640.1	Salmonella phage Segz_1
NC_054641.1	Salmonella virus KFS-SE2
NC_054642.1	Salmonella phage Seszw_1
NC_054643.1	Salmonella phage SeSz-2
NC_054644.1	Salmonella virus VSt472
NC_054645.1	Salmonella phage LPST10
NC_054646.1	Salmonella phage VB_StyS_BS5
NC_054647.1	Salmonella phage Akira
NC_054648.1	Salmonella phage vB_Se_STGO-35-1
NC_054649.1	Escherichia phage vB_EcoS_swi2
NC_054650.1	Shigella phage DS8
NC_054651.1	Escherichia phage C1
NC_054652.1	Klebsiella phage YX3973
NC_054653.1	Klebsiella virus KpV2811
NC_054654.1	Klebsiella phage vB_KpnS_ZX4
NC_070772.1	Vibrio phage Seahorse
NC_070773.1	Vibrio phage NF
NC_070949.1	Erwinia phage Midgardsormr38
NC_071003.1	Enterobacter phage vB_EclS_CobraSix
NC_071142.1	Klebsiella phage VLCpiS13d
NC_071144.1	Klebsiella phage VLCpiS13f
NC_071145.1	Klebsiella phage VLCpiS13e
NC_071146.1	Klebsiella phage vB_KpnS_MK54
NC_071147.1	Klebsiella phage KP591P1
NC_071148.1	Klebsiella phage KP591P3
NC_071149.1	Klebsiella phage vB_Kpn_ZCKp20p
NC_071150.1	Klebsiella phage ZCKP8
NC_071151.1	Klebsiella phage vB_Kpn_ZC2
NC_071152.1	Klebsiella phage VLCpiS13a
NC_071153.1	Klebsiella phage BUCT610
NC_071154.1	Klebsiella phage BUCT541
NC_071155.1	Klebsiella phage VLCpiS13b
NC_071156.1	Klebsiella phage 6991
NC_071157.1	Klebsiella phage VLCpiS13c
JF314845.1	Cronobacter phage ES2

Table S4. Sequences of the oligonucleotide primers used in this study. These were used to complete the mEp021 genome by sequencing the gaps (GS) between assembled contigs; to validate the mEp021 integration site (*att* site); for PCR amplification (PCR) of the *gp*15 (Rep_{021}), *gp*81 (Lpp_{021}), and *gp*116 (Lpp_{010}) genes respectively cloned into the pKQV4 expression vector, and to verify the Keio mutant strains (KMV) *omp*A^- and *lam*B^-, as indicated in the third column.

Primers	Sequence 5´ 3´	Use in this study
1fwd	ccctggctttcaagatattcccgcg	GS
1rev	gatgtacgcaatagtgacggacggg	GS
2fwd	tatcttaaagcaccgccaccgccac	GS
2rev	ttcctgcctccttcctttgattcgc	GS
3fwd	cgcgatatttgcgcatacgtggatc	GS
3rev	ggtaatgacctccgaagacctcacc	GS
4fwd	ccgttccggtgttgtttacggtcac	GS
4rev	tcctgccttgtcgttgtagaaggac	GS
5fwd	tcaggtagtggcacaaagcaaccgg	GS
5rev	aattataggcgtggtggttgatgcg	GS
6fwd	tacccgtgcctgtaacagtgttgcc	GS
6rev	tgtgccgccttcgttcatctgttgc	GS
7fwd	cggaacaggatcaaggattgtcagg	GS
7rev	accaccagaatagccgcaacagcg	GS
8fwd	tttggcccttgctttaacattgcgc	GS
8rev	gctgggtgaaattacaggtggcgac	GS
T1	ttcttccccttcgctaatagctc	*att*L
B1	agacggaatagggtccggttc	*att*R
T2	tatctccgctatacctcaatcac	*att*R
B2	tcaatgattacgcacaacttc	*att*L
Rep021 Fwd	ccggaattcatgaaaacaaaatggtatgacttagc	*gp*15 PCR
Rep021 Rev	cccaagcttatggcaggtttacgatcttcgcatc	*gp*15 PCR
Lpp021 Fwd	cgcggcggaattcatgaaaatggtaacaattatcgctg	*gp*81 PCR
Lpp021 Rev	tcaagcttttagtgatggtgatggtgatgtccactaccttwgtaccactccttacttc	*gp*81 PCR
Lpp010 Fwd	gaattcatgaaaaacgtgttcaaag	*gp*116 PCR
Lpp010 Rev	aagcttcttttagtagttggctacac	*gp*116 PCR
Kan Fwd	ggccagatctgatcaagaga	KMV
*lam*B Rev	ccggcccaggttttgctatt	KMV
*omp*A Rev	ttaagcctgcggctgagt	KMV

Table S5. Viral genome clusters (VGC) obtained through VirClust analysis**.** The PC-based intergenomic distance tree was split in viral genome clusters using a distance threshold of 0.7. A total of 110 genomes were included: 48 corresponding to the mEp_{immI} group, 11 reference siphovirus (c2, HK022, HK97, L5, Lambda, N15, phi-C31, psiM2, SPBc2, T1, T5),1 reference podovirus (P22) and 50 phages that were neighbors to the mEp_{immI} branch in the ViPTree analysis.

genome_cluster_ID	genome_name	length	silhouette_width
**1**	ctx8n3	58116	**0.73**
**1**	mEp515	53768	**0.74**
**1**	RM9245	55393	**0.75**
**1**	mEp021	54655	**0.75**
**1**	E21845	56040	**0.77**
**1**	FBP1	55841	**0.77**
**1**	Ecol_244	55379	**0.78**
**1**	MS21_1	57244	**0.78**
**1**	STEC306	55377	**0.78**
**1**	STEC307	55378	**0.78**
**1**	STEC308	55378	**0.78**
**1**	mEp013	53046	**0.78**
**1**	GN02175	53924	**0.79**
**1**	HVH110	53651	**0.79**
**1**	KKa019	53525	**0.79**
**1**	1_110_08_S4_C1	54039	**0.8**
**1**	1_392_07_S4_C3	54510	**0.8**
**1**	504005_aEPEC	54703	**0.8**
**1**	G180	54727	**0.8**
**1**	LSU61	53287	**0.8**
**1**	PAR	53068	**0.8**
**1**	UMNK88	55827	**0.8**
**1**	0621_18038ec	56309	**0.81**
**1**	127ec	55081	**0.81**
**1**	ATCC23502	55207	**0.81**
**1**	HVH103	54400	**0.81**
**1**	M1_5	55216	**0.81**
**1**	RHBSTW_00392	54598	**0.81**
**1**	WCHEC025970	54864	**0.81**
**1**	09_00049ec	53858	**0.82**
**1**	190ec	55205	**0.82**
**1**	2_331ec	54383	**0.82**
**1**	FHI99	56458	**0.82**
**1**	GN05505	54276	**0.82**
**1**	KOEGE62	55088	**0.82**
**1**	KTE83	54775	**0.82**
**1**	UMEA3323_1	55169	**0.82**
**1**	ct11k1	54581	**0.82**
**1**	ctPXR1	55155	**0.82**
**1**	FAH	54748	**0.83**
**1**	GN04592	54442	**0.83**
**1**	HVH69	54935	**0.83**
**1**	MIN12	55179	**0.83**
**1**	MLI107	54793	**0.83**
**1**	mEp010	53876	**0.83**
**1**	mEp044	54642	**0.83**
**1**	mEp554	53941	**0.83**
**1**	C16EC0292	54622	**0.84**
2	VLCpiS13d	46281	0.43
2	VLCpiS13c	47997	0.45
2	vB_EcoS_Sa179lw	46833	0.45
2	Akira	45367	0.49
2	Sf11SMD_2017	46454	0.49
2	VLCpiS13b	48190	0.49
2	C1	46667	0.5
2	YX3973	46907	0.5
2	vB_Se_STGO_35_1	47483	0.5
2	KpV2811	46391	0.51
2	Segz_1	48285	0.51
2	DS8	44605	0.52
2	IME207	47564	0.52
2	KP591P1	45696	0.52
2	KP591P3	45716	0.52
2	Klebsiella_6991	46373	0.52
2	SeSz_2	45049	0.52
2	VLCpiS13f	48050	0.52
2	vB_SenS_SB2	45126	0.52
2	64795_sal3	45342	0.53
2	BUCT541	46100	0.53
2	BUCT610	46774	0.53
2	E1	45051	0.53
2	vB_KpnS_MK54	46218	0.53
2	KFS_SE2	48608	0.54
2	VLCpiS13e	47720	0.54
2	ZX4	45424	0.54
2	vB_EcoS_swi2	47611	0.54
2	VB_StyS_BS5	47604	0.55
2	VLCpiS13a	47956	0.55
2	pYD38_A	47552	0.55
2	LPST10	47657	0.56
2	VSt472	46905	0.56
2	ZCKP2	48258	0.56
2	ZCKP8	48490	0.56
2	pIS4_A	47624	0.56
2	vB_Kpn_ZCKp20p	48797	0.56
2	Seszw_1	45881	0.57
2	Skate	47393	0.57
3	c2	22172	0
4	ENT47670	47611	0
5	SPN3UB	47355	0.21
5	ES18	46900	0.27
5	vB_SosS_Oslo	49116	0.27
6	ES2	22162	0.03
6	vB_EclS_CobraSix	47816	0.05
7	HK97	39732	0.54
7	HK022	40751	0.57
8	L5	52297	0
9	Lambda	48502	0.1
9	N15	46375	0.21
10	Midgardsormr38	50485	0
11	NF	44507	0.39
11	Vibrio_phage_Seahorse	45171	0.41
12	P22	41724	0
13	phi_C31	41491	0
14	psiM2	26111	0
15	SPBc2	134416	0
16	T1	48836	0
17	T5	121750	0
18	vB_EhrS_49	46835	0.1
18	vB_EhrS_59	47116	0.22

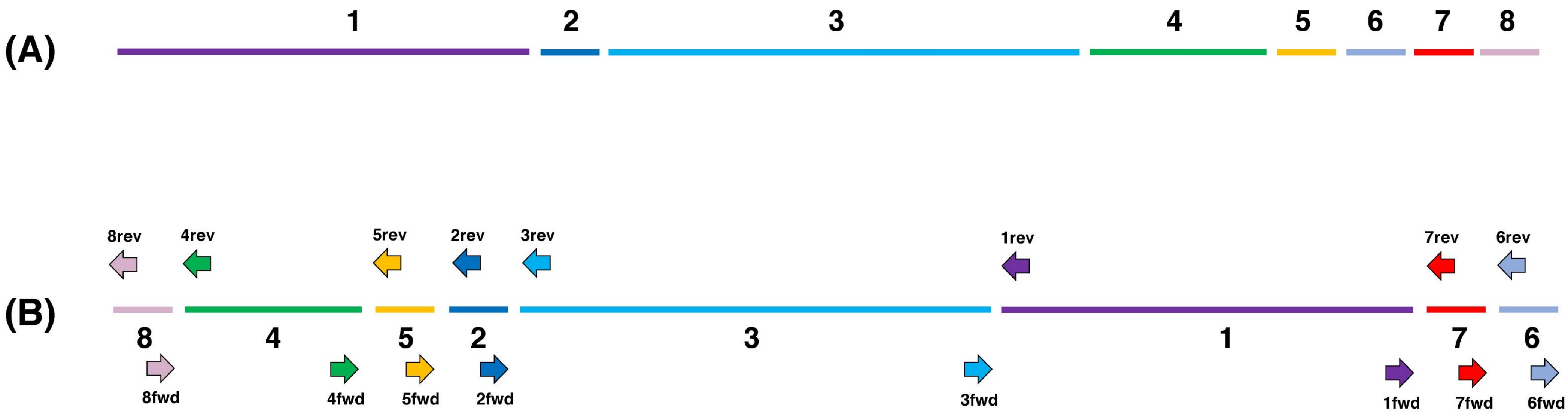

Figure S1. Assembly strategy used to complete the mEp021 genome sequence. **(A)** The color lines represent the unconnected contigs assembled from SOLiD sequencing reads (see Materials and Methods). **(B)** The actual order of contigs in the mEp021 genome was determined by chromosome walking by Sanger sequencing, using primers amplifying outwards of each assembled contig (colored arrows). The complete genome of mEp021 was estimated to be 54,655 bp.

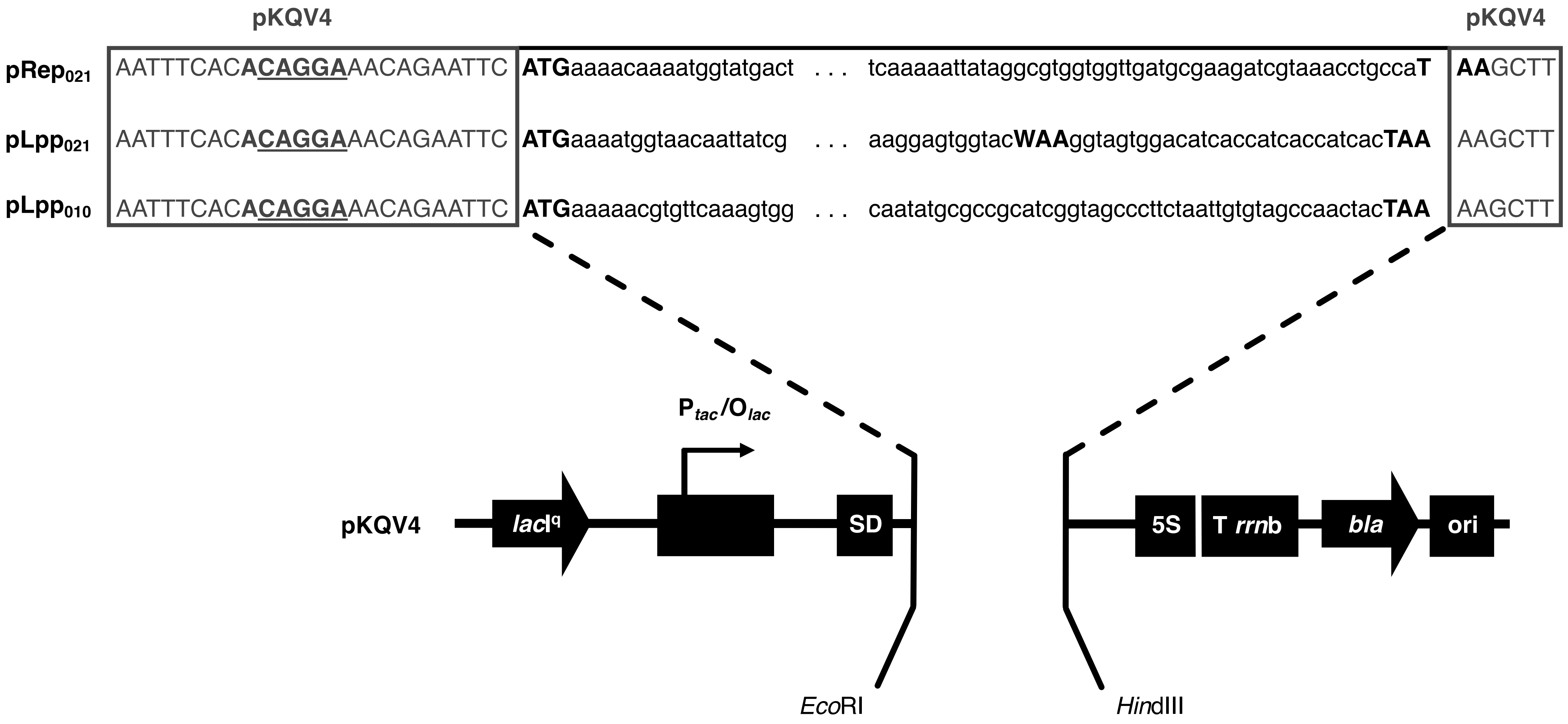

Figure S2. Cloning of phage lipoproteins and the repressor genes in pKQV4 expression vector. The PCR products of the *gp*15 and *gp*81 genes, which respectively code for the lipoprotein Lpp_{021} and the Rep_{021} repressor of phage mEp021, were inserted into the low copy pKQV4 expression vector using the *Eco*RI and *Hin*dIII restriction sites. The *gp*116 gene coding for the lipoprotein Lpp_{010} of phage mEp010 was cloned likewise. Vector sequences are depicted in upper-case font, the Shine-Dalgarno (SD) region is underlined, and viral sequences are in lower-case; start and stop codons are indicated in upper-case bold font.

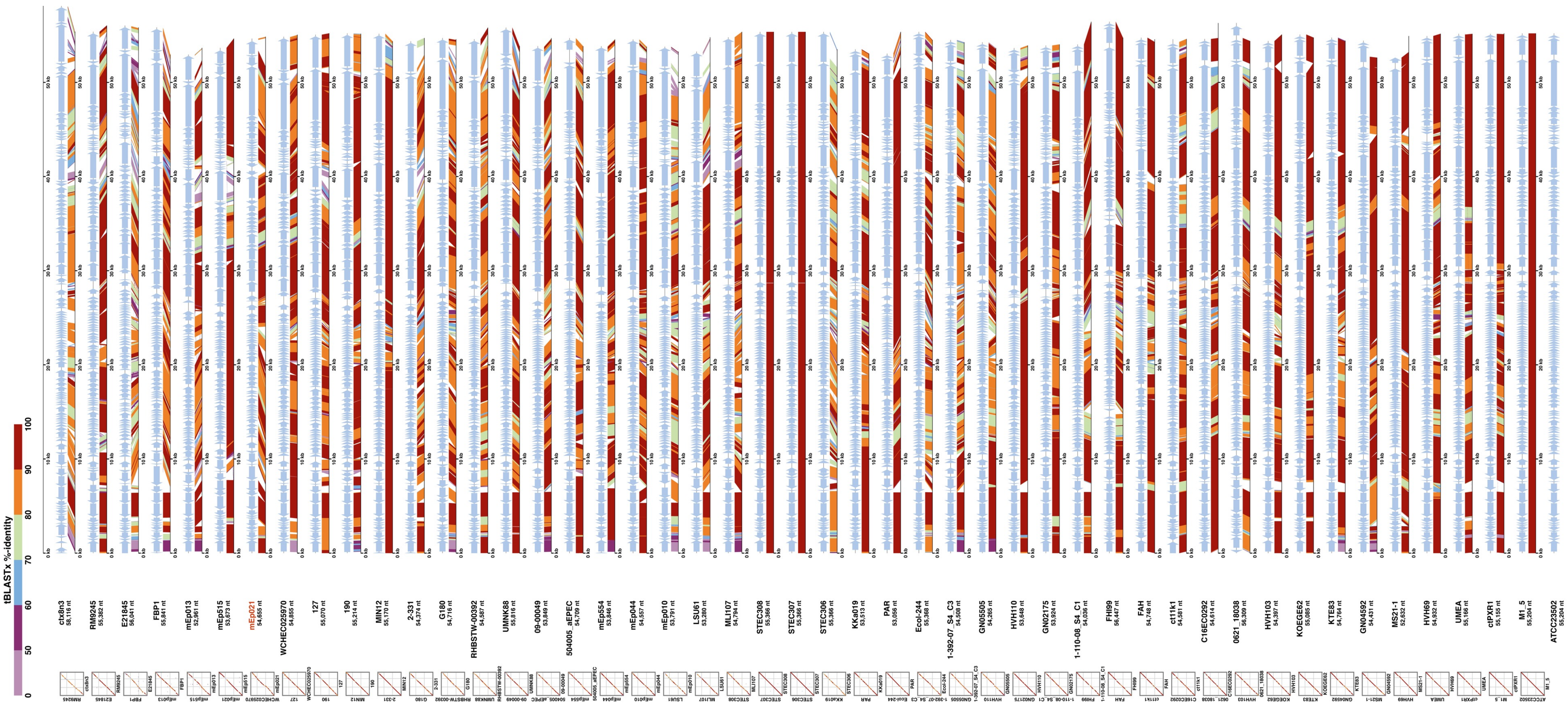

Figure S3. Alignment of the 48 bacteriophages and prophages of the mEp$_{immI}$ group reveals high synteny. The predicted proteins of each complete genome were aligned using the ViPTree 4.0 server. The color bar on the left indicates the identity percentage values, computed by tBLASTx. Graphical representations of the pairwise alignments of adjacent phages are included at the bottom, showing high correlation in all cases.

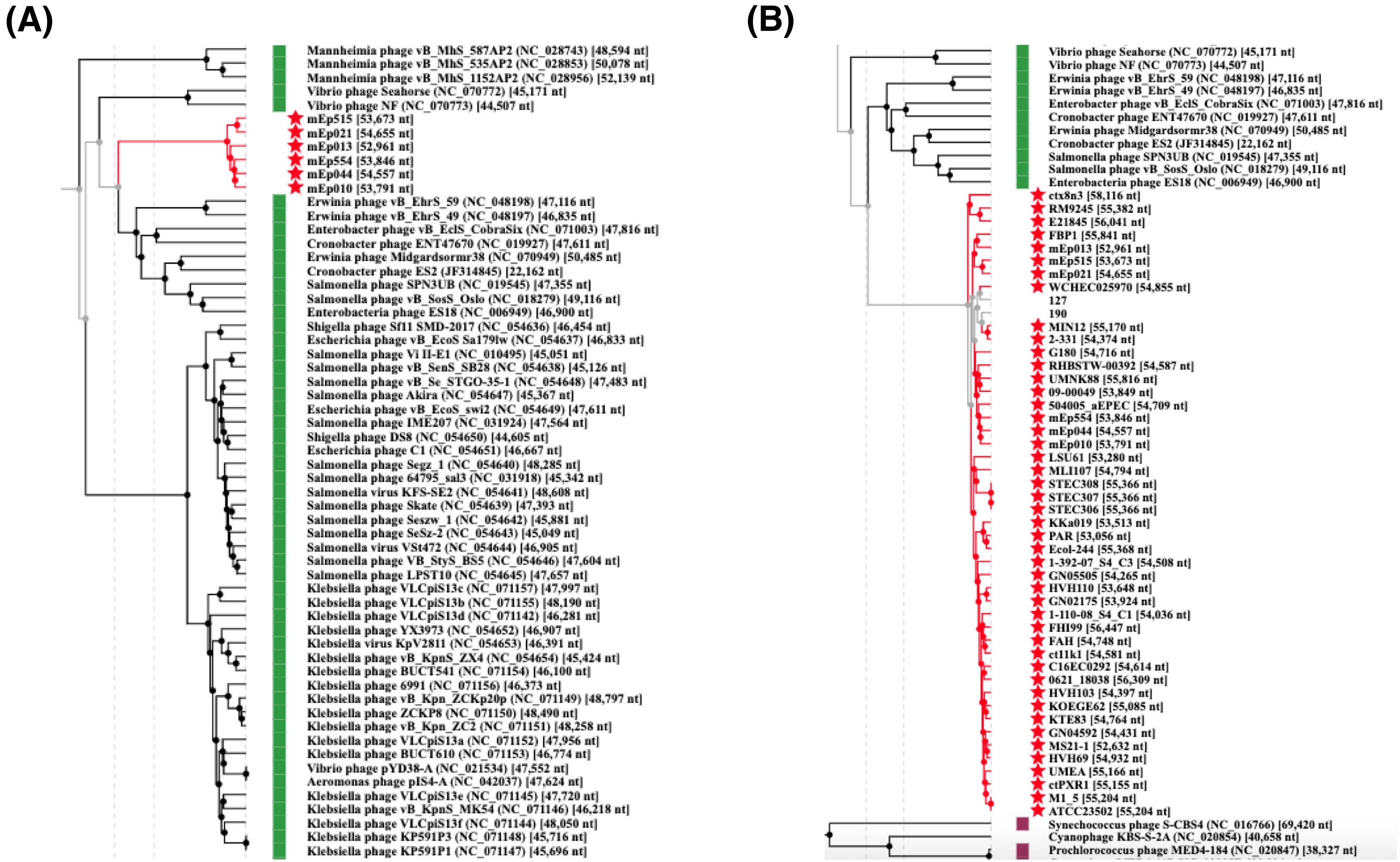

Figure S4. Proteome-based analysis of the mEp_{imml} phages. **(A)** The whole proteome of the six mEp_{imml} phages sequenced in this work was compared to other >5600 proteomes from dsDNA phages using the ViPTree 4.0 server. Expanded view of the mEp_{imml} cluster from the proteomic tree shown in Figure 2C. The mEp_{imml} phages form a separate cluster, indicated by red lines. **(B)** The 42 homologous phages and prophages were incorporated to the analysis, revealing that the total mEp_{imml} group (n=48) remains as compact and separate phylogenetic branch in the proteomic tree, displaying notable closeness among its members.

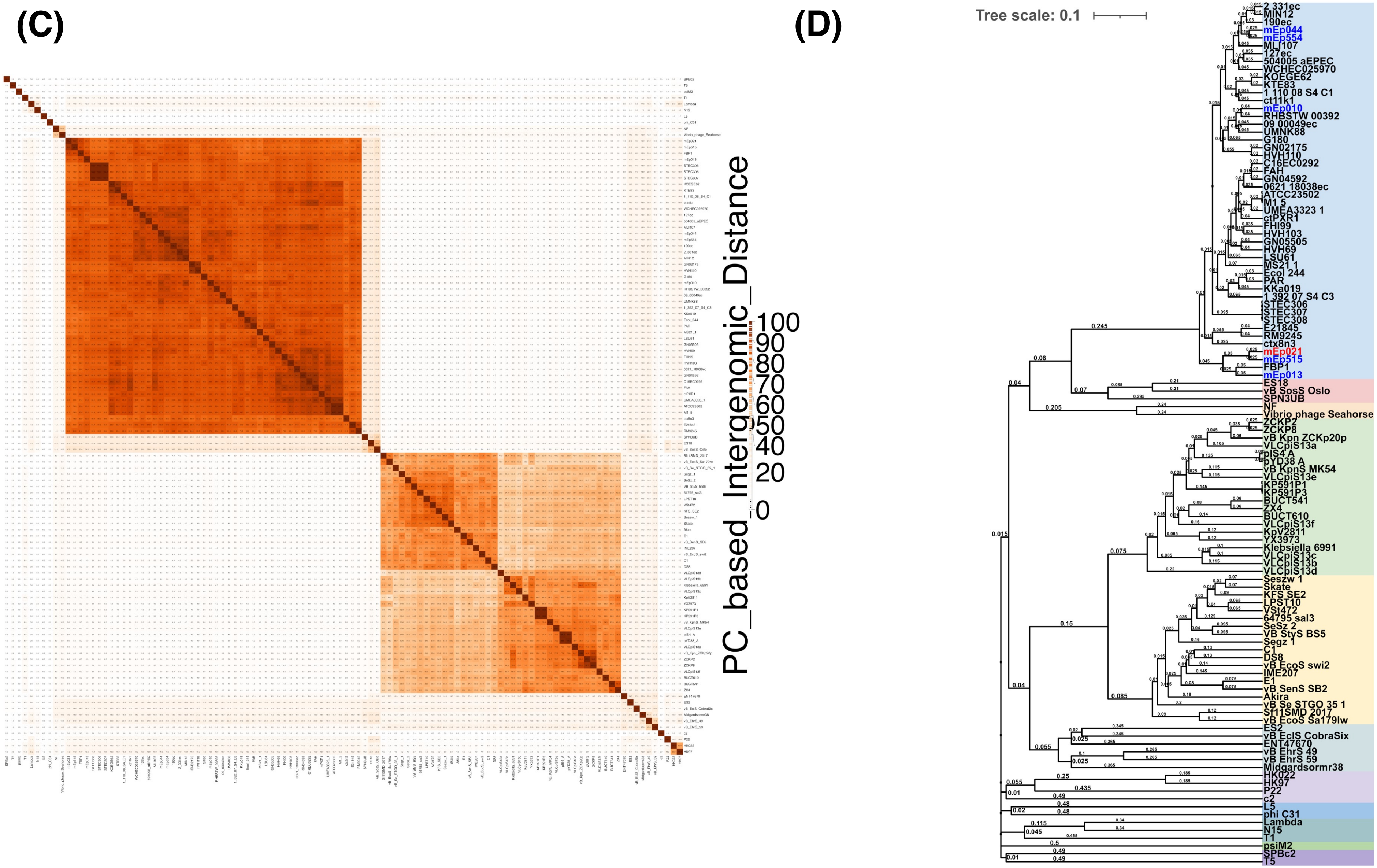

Figure S4 (continued). **(C)** VirClust analysis of the 48 mEp_{immI} genomes and 110 phage genomes including 12 reference phages and 50 phages observed as neighbors in the ViPTree analysis. The heatmap shows the pairwise intergenomic similarity values calculated from the identified protein clusters (PC), where dark orange color represents higher similarity values. Evident clustering of the mEpimmI phages is observed. genome clusters (VGCs). **(D)** Hierarchical clustering of the phage genomes based on their PC-calculated intergenomic distances; bootstrap probability (BP) values are indicated (100 Bootstrap replicates); the newick file was visualized with online tool iTol (https://itol.embl.de/upload.cgi).

(A)

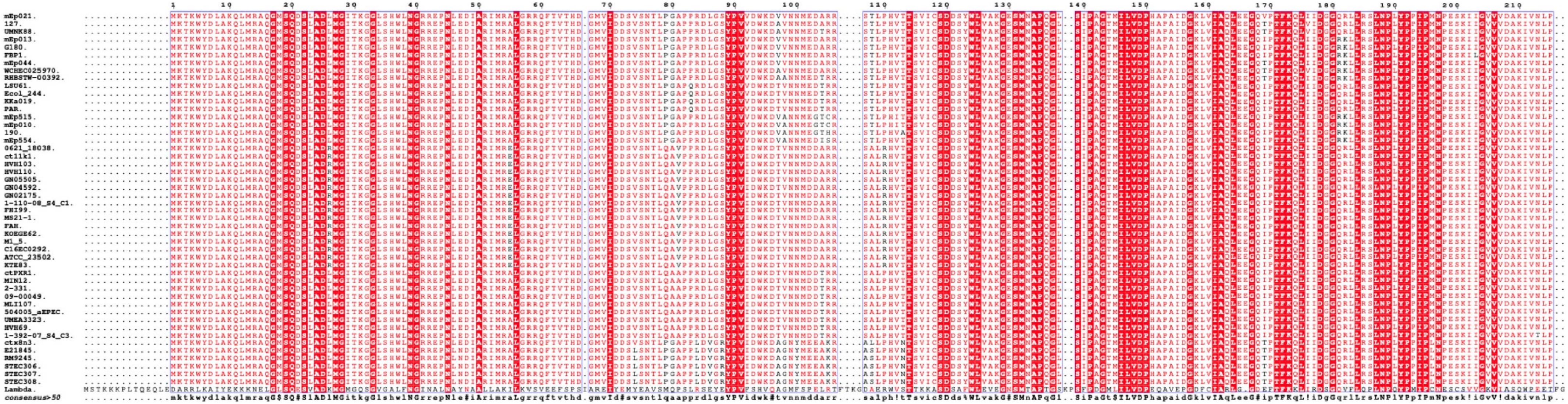

Figure S5. Phylogenetic analysis of the repressor proteins of the mEp_{immI} phages and prophages. **(A)** Amino acid sequence alignment of the predicted repressor proteins of 48 phages and prophages, revealing almost complete sequence identity among them. The CI repressor of phage λ was included as an external reference (bottom).

(B)

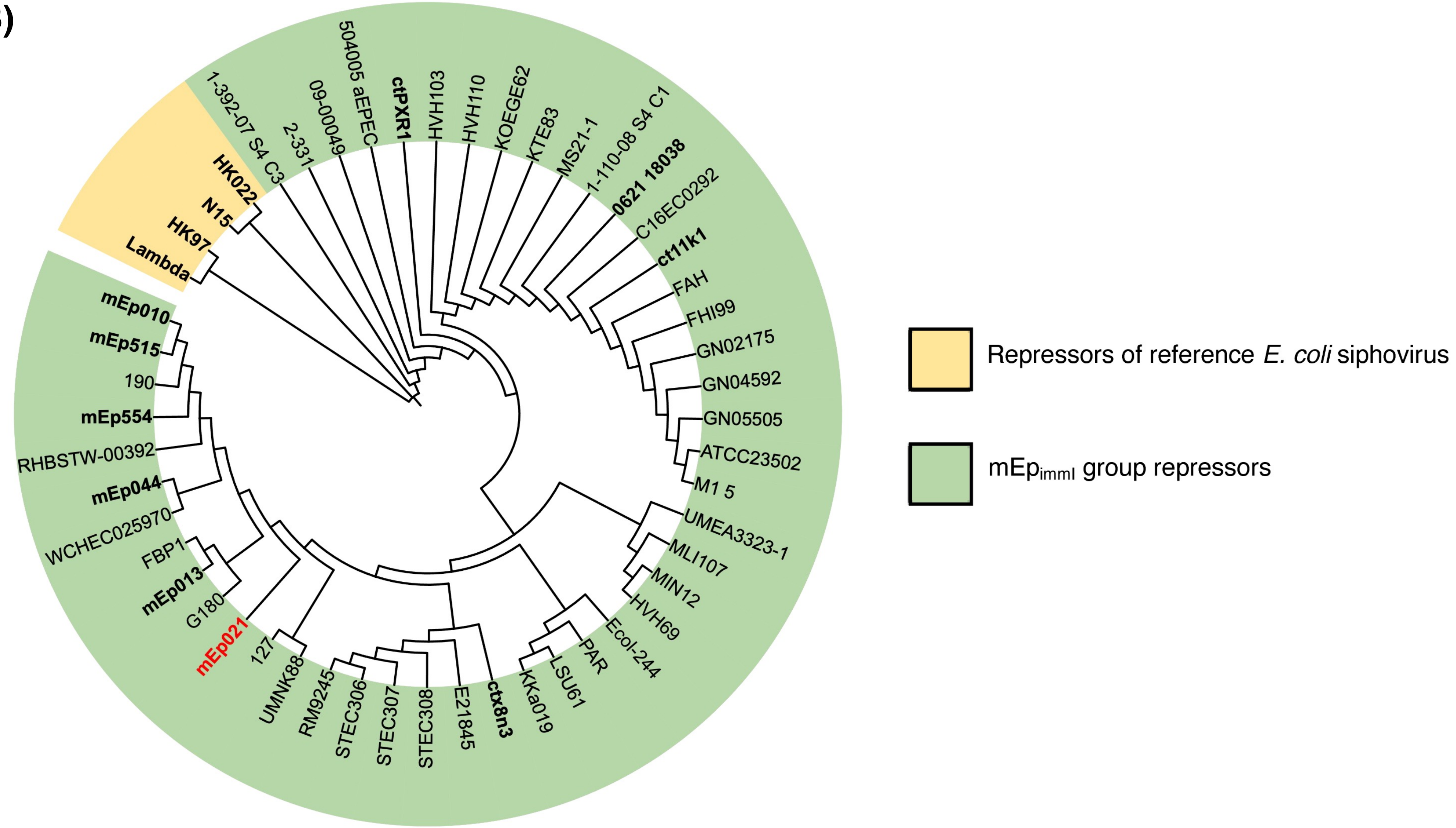

Figure S5 (continued). (B) Rooted phylogenetic tree of the 48 predicted repressor proteins of the mEp$_{immI}$ group using the NGPhylogeny.fr server; the divergence between these branches is minimal. Repressor proteins of reference *E. coli* siphovirus (Lambda, HK97, N15, and HK022) were included as outgroup, and these formed separate branches as expected.

(A)

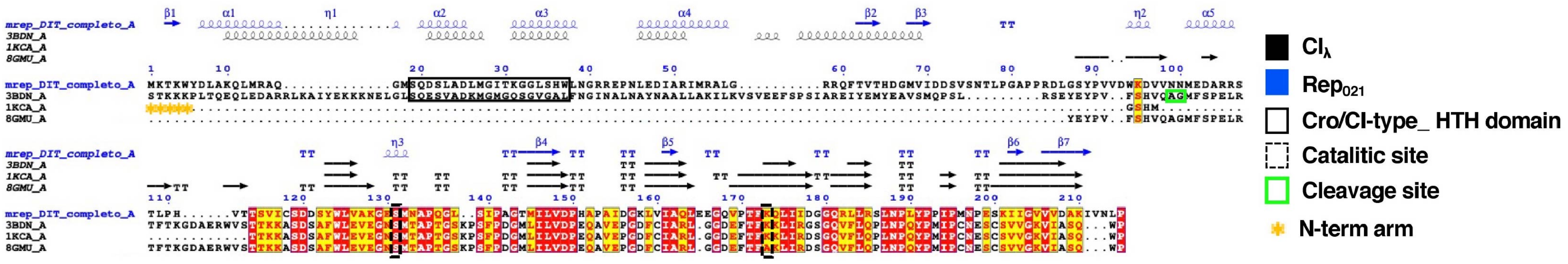

Figure S6. Structural comparison of the mEp021 and l repressor proteins. **(A)** Amino acid sequence alignment of Rep_{021} from phage mEp021 (indicated at the top with blue label) and the CI repressor from phage Lambda (in black). The amino acid sequences of the latter correspond to the crystallographic structures 3BDN, 1KCA, and 8GMU (as indicated on the left side of the alignment figure). Secondary structures (alpha helices and beta folds) are indicated at the top of the alignment, using the same color code. Functional regions include the HTH domain (conformed by alpha helices 2 and 3), the catalytic site and the cleavage site, which are illustrated in colored boxes as indicated in the right panel. These domains share a similar location in both proteins. Additional operator-binding amino acids of CI_{λ} are indicated with yellow asterisks. Despite the low amino acid sequence similarity in the N-terminal region, a notable conservation was observed in the C-terminal region of both proteins.

(B)

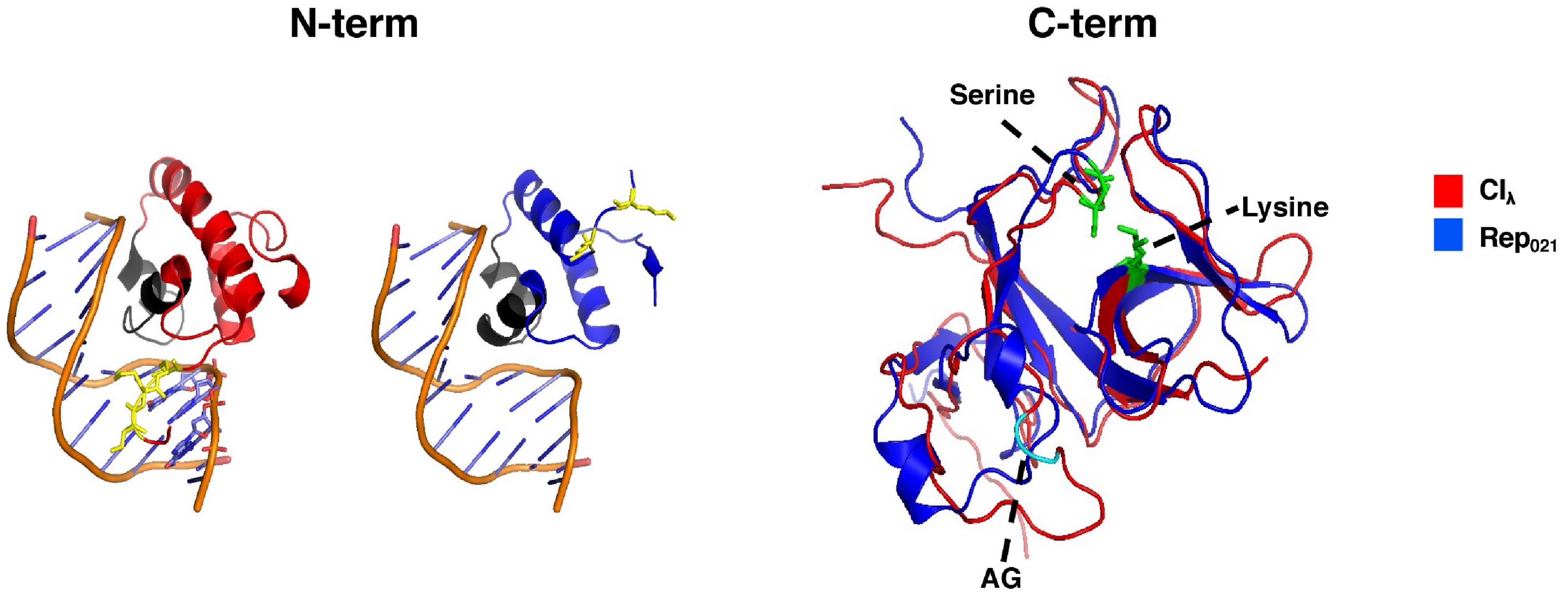

Figure S6 (continued). Structural comparison of the mEp021 and l repressor proteins. **(B) Left panel:** The N-terminal segment of the Rep_{021} (in red) contains the HTH DNA binding motif, which is structurally similar to that of the CI_{λ} protein (blue). **Right panel:** Superposition of the C-terminal segments of both repressor proteins, containing the LexA-like domain. The CI_{λ} catalytic site for self-cleavage is shown in green and the AG cleavage site is shown in light blue.

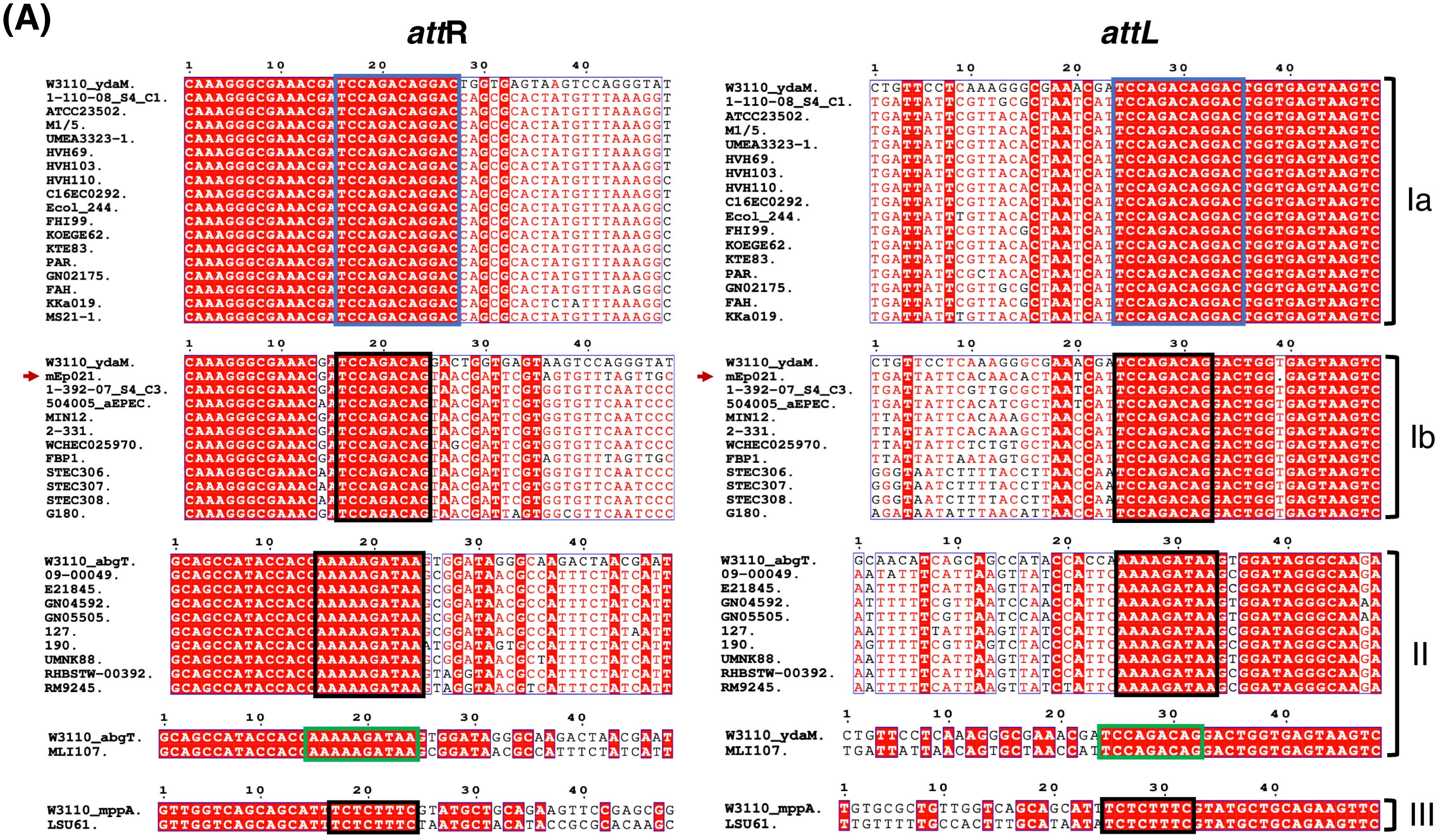

Figure S7. Predicted integration sites and integrase protein types of the mEp$_{immI}$ group. **(A)** Alignment of the integration regions of 38 mEp$_{immI}$ homologous prophages in their host genomes. Lysogen sequences upstream and downstream of the integrated prophage genomes were included in the alignments, and are displayed as the most conserved regions (red background). The sequence at the top of each group corresponds to the W3110 genome; left panels correspond to the left attachment sites (*att*L) and the right panels to the right attachment sites (*att*R) of the prophages. According to sequence motifs (indicated in boxes), four distinct integration sites were identified: Ia, Ib, II and III (indicated by brackets at the right). The mEp021 prophage sequence is indicated by a red arrow at the left. The green box indicates a putative host DNA deletion.

(B)

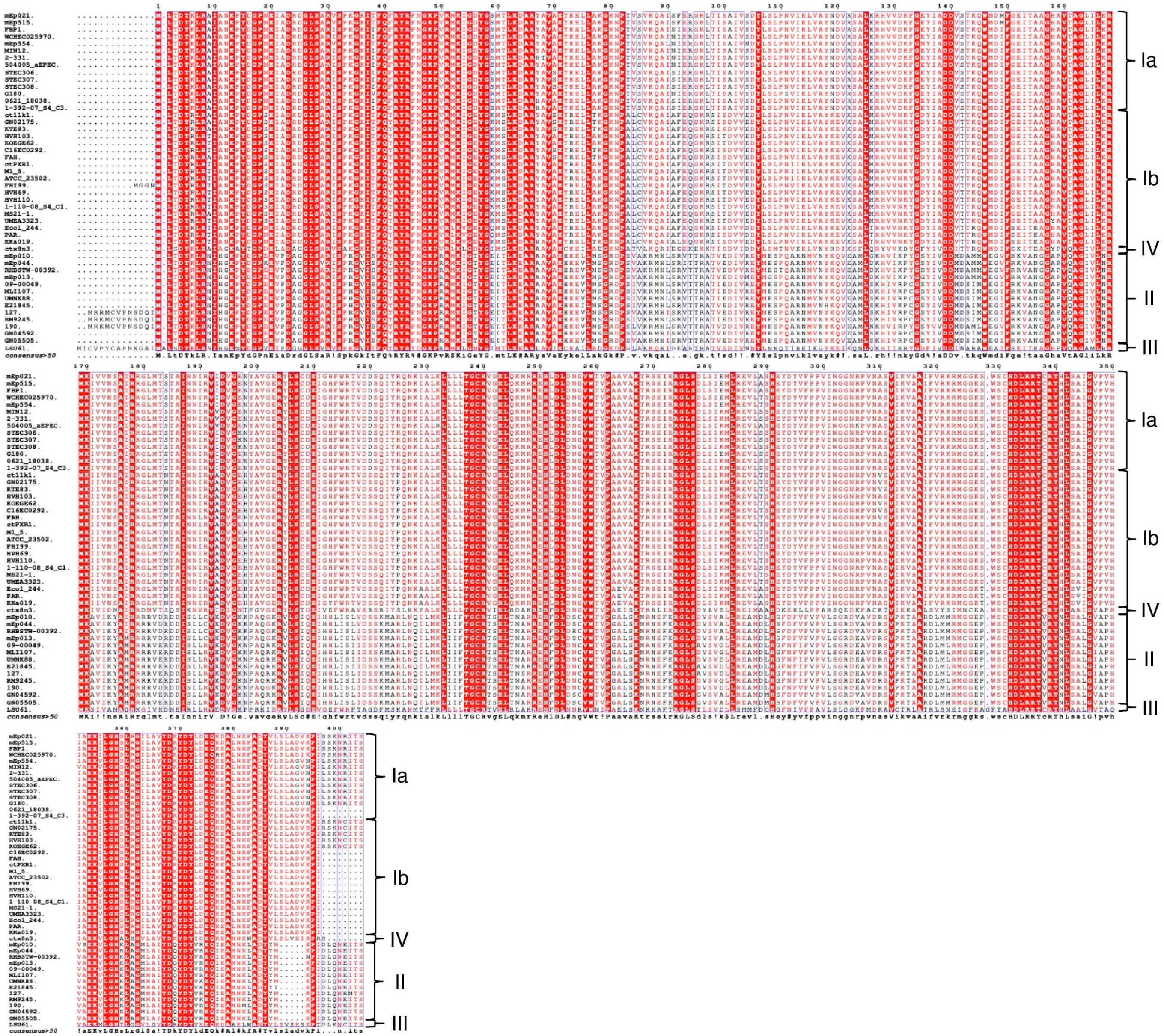

Figure S7 (continued). (B) The amino acid sequence alignment of the integrase proteins of the 48 phages and prophages shows that they can be classified into five sequence-types (Ia, Ib, II, III, and IV as indicated at the right of the figure). The sequences between groups Ia and Ib are very similar, while the sequences of group II display differences starting from position 84; groups III and IV include only one sequence, respectively.

(C)

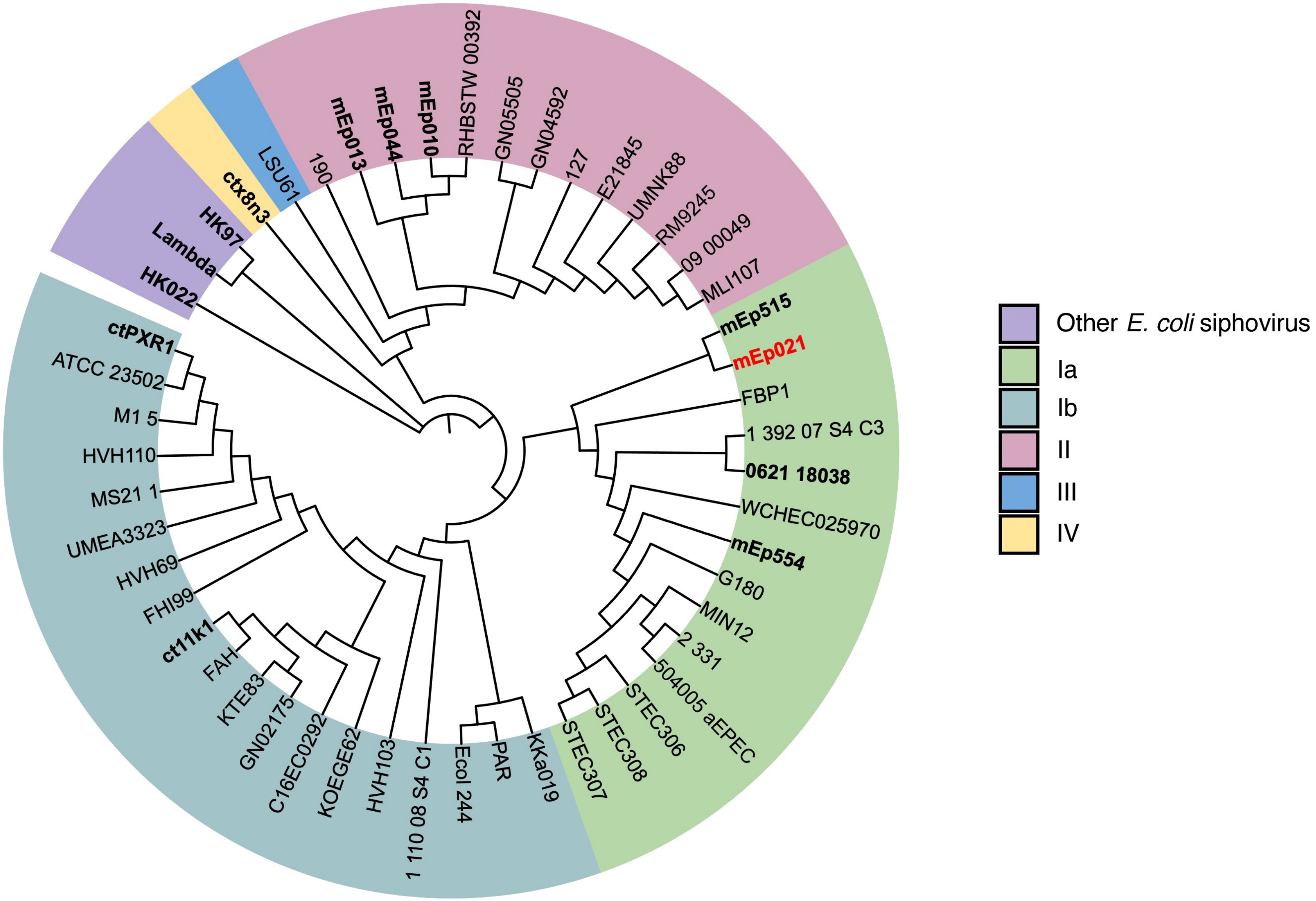

Figure S7 (continued). (C) Phylogenetic tree of the 48 integrases of the mEp$_{immI}$ group. According to amino acid sequences, four groups of integrases are well defined, as indicated by the color code at the right. mEp021 is indicated in red font. The integrases of the lambdoid phages HK97, HK022, and Lambda form a separate cluster, indicated in light purple background.

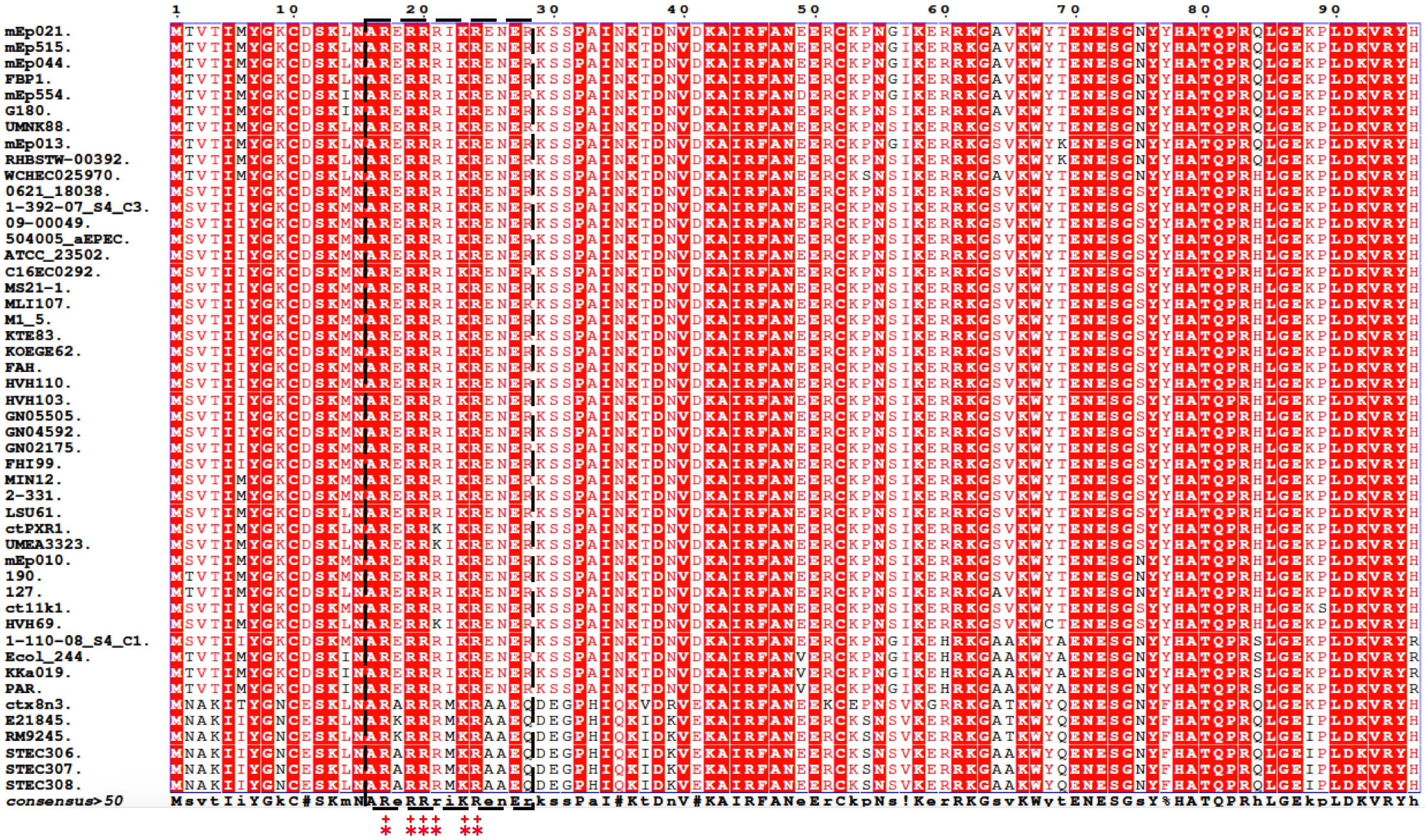

Figure S8. Conserved Gp17 (N_λ-like) proteins in the 48 mEp_{immI} phages and prophages. Alignment of Gp17 predicted proteins from the 48 phages and prophages of the mEp group. The arginine-rich motif (ARM) is strongly conserved and is indicated by the black dashed box; all arginines at positions 17, 19, 20, and 24 were fully conserved. The residues of the N_l-like proteins that are required for boxB loop-binding are indicated by red asterisks; polar basic amino acids are indicated (+).

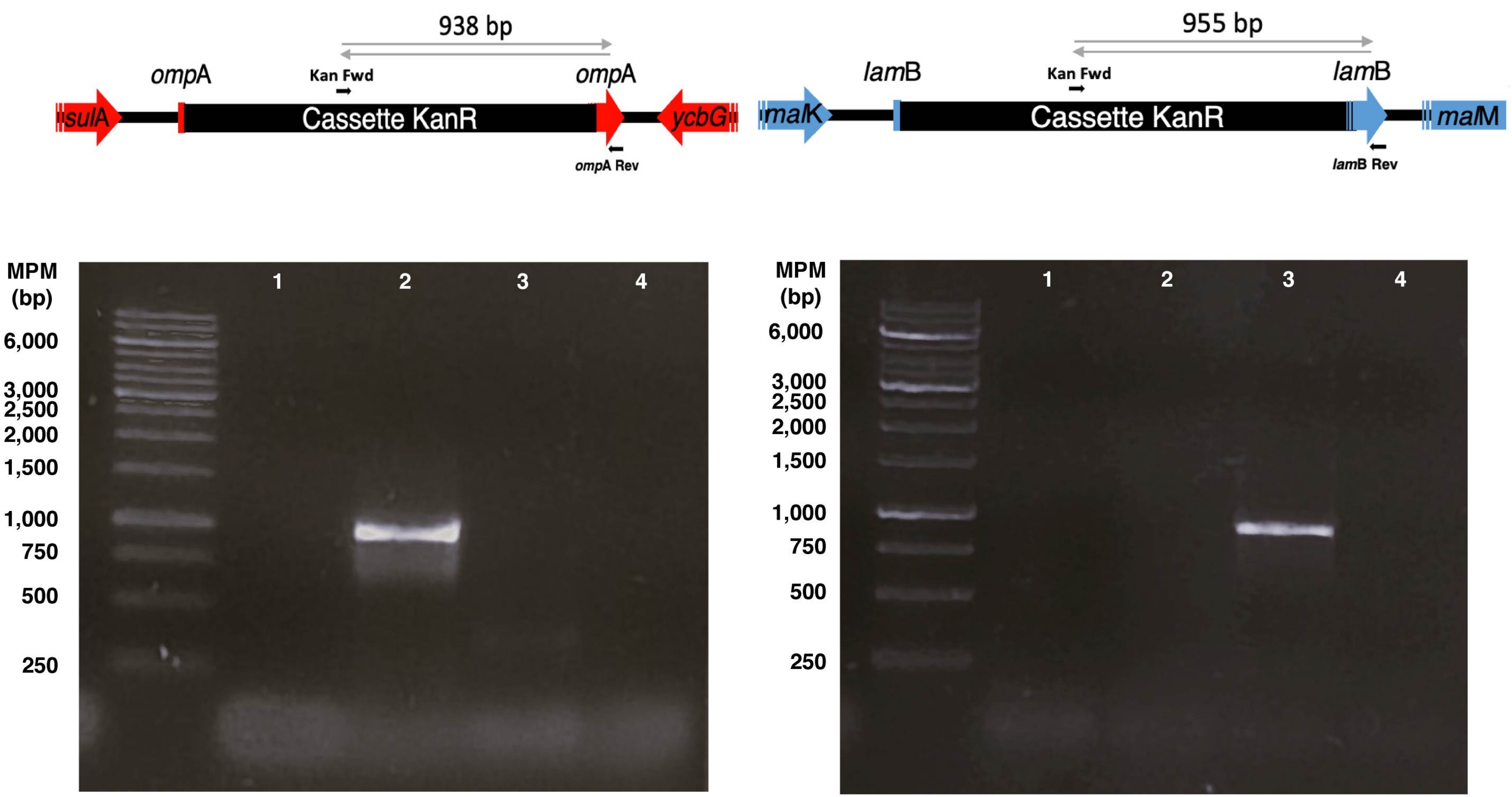

Figure S9. PCR validation of the Keio mutants: *omp*A⁻ and *lam*B⁻ (left and right panels, respectively). Oligonucleotide primer pairs were designed to verify the insertion of the kanamycin resistance gene, which disrupts the distinct membrane protein coding genes. A common forward primer was aimed at the Kan gene (Kan Fwd) and two different reverse primers were designed to target the 3' coding regions of the *omp*A and *lam*B genes, respectively (as indicated at the top of the figure). Different DNA templates were used in the PCR reactions: Lanes: 1, W3110; 2, JW3991-1 (*lam*B⁻); 3, JW0940-6 (*omp*A⁻); 4, non-template control. The expected 938 bp and 955 bp amplicons were observed at the corresponding lanes, confirming the genotype of the *lam*B⁻ and *omp*A⁻ mutant strains of the Keio collection.

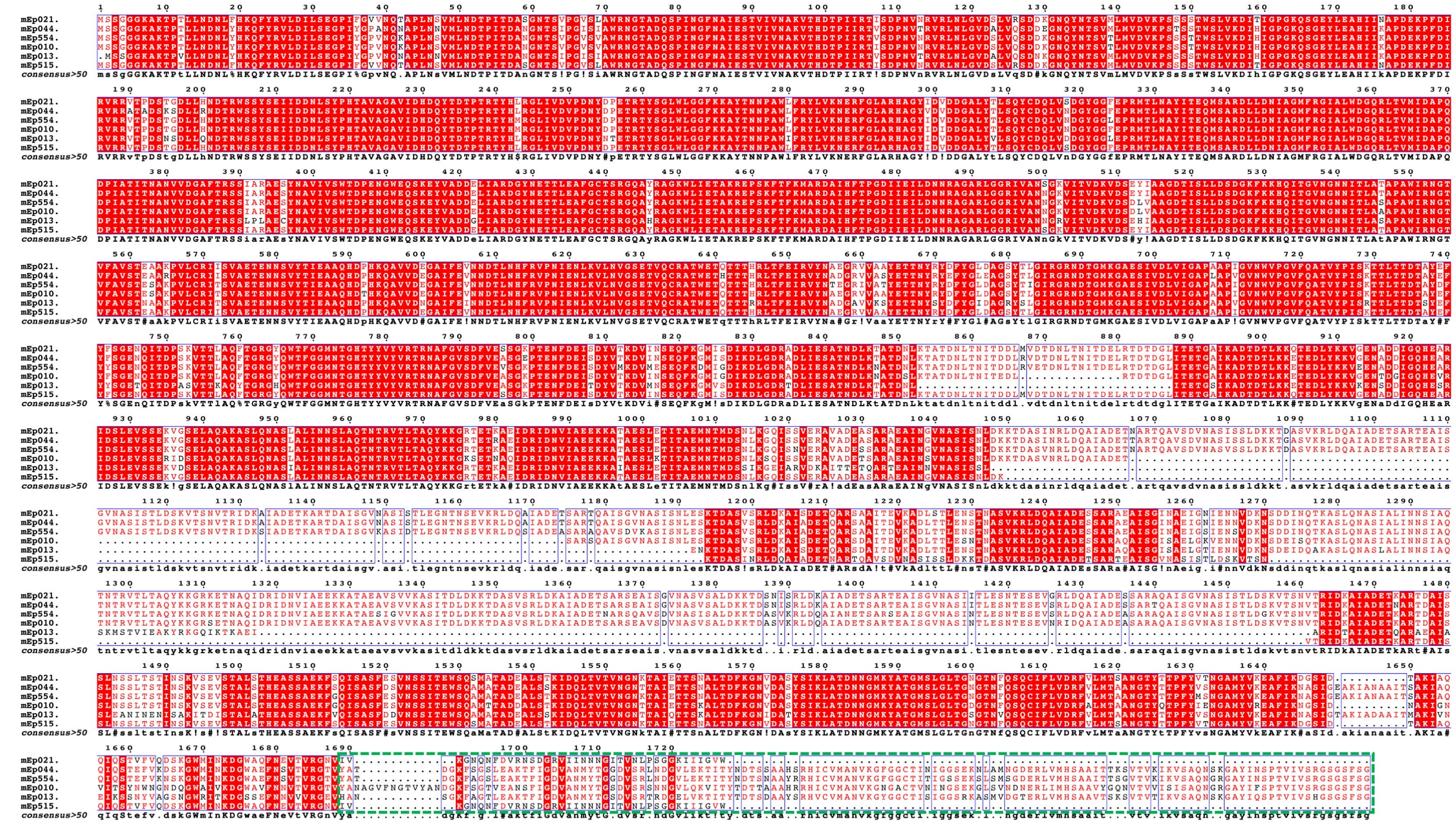

Figure S10. Comparison of the J proteins of mEp_{immI} phages, related to the recognition of the bacterial outer membrane receptor (OMR). Amino acid sequence alignment of six predicted J proteins from representative mEp_{immI} phages shows that sequence variations mainly occur at the C-terminal portion. The differences observed at the C-terminal end of these proteins, which contain the putative receptor binding domain (green box), could account for the use of specific bacterial receptor proteins.

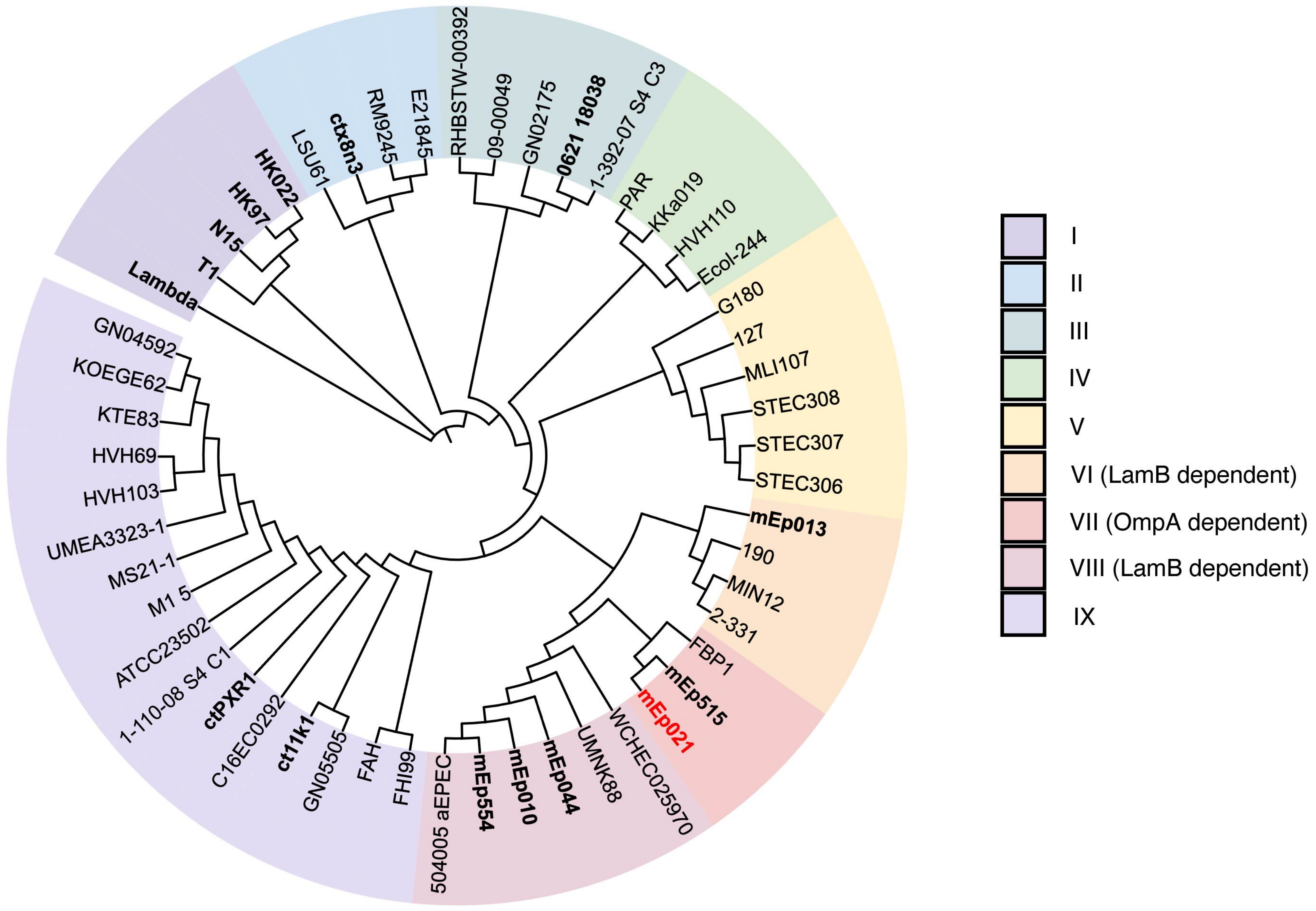

Figure S11. Phylogenetic tree based on the J protein alignments of 53 mEp_{immI} phages and prophages. The main phylogenetic clusters are assorted in colored background, as indicated. Consistently, the phages that require the host receptor protein OmpA are phylogenetically closer (mEp021 and mEp515, as described in Figure 2B), and those that require the LamB receptor are grouped in separate clusters; the presence of other phylogenetic clusters (i.e. J protein variants) suggests the use of diverse host receptor proteins.
